# Alkali Metalation Enables Natural Anthraquinone Derivatives as Sustainable Cathode Materials for Lithium‐Ion Batteries

**DOI:** 10.1002/advs.202513052

**Published:** 2025-08-30

**Authors:** Xinyue Zhu, Xianlong Zhou, Lingchao Cai, Thomas Heine, Yu Jing

**Affiliations:** ^1^ Jiangsu Co‐Innovation Centre of Efficient Processing and Utilization of Forest Resources, College of Chemical Engineering Nanjing Forestry University Nanjing 210037 China; ^2^ T TU Dresden Fakultät für Chemie und Lebensmittelchemie Bergstraße 66c 01062 Dresden Germany; ^3^ Center for Advanced Systems Unerstanding (CASUS) Helmholtz‐Zentrum Dresden‐Rossendorf Am Untermarkt 2 02826 Görlitz Germany; ^4^ Department of Chemistry and ibs‐cnm Yonsei University Seodaemun‐gu Seoul 120‐749 Republic of Korea

**Keywords:** anthraquinones, carbon nanotubes, electrode materials, lithium‐ion batteries, molecular polarity

## Abstract

Organic electrode materials (OEMs) derived from natural quinones can enable sustainable lithium‐ion batteries (LIBs) if their dissolution‐induced capacity fading in organic electrolytes and their conductivity issues are addressed. It is demonstrated that converting natural anthraquinones (AQs) into organic alkali‐metalated salts effectively inhibits their dissolution in aprotic electrolytes. For this purpose, a solubility indicator (ΔMPI) is developed, which reliably guides the selection of compatible OEMs and electrolytes. The effect of alkali metalation (i.e., replacing ─OH with ─OM, M = Li, Na, K) on the physicochemical properties of AQs is revealed, and validate experimentally the suppressed dissolution and improved cyclic stability of metalated AQs. Finally, the issue of low conductivity is solved by the addition of carbon nanotubes (CNTs). The best device, potassiated 2,6‐DHAQ with added CNTs (K_2_(2,6‐DHAQ@CNTs)), exhibits an excellent electrochemical performance, maintaining a capacity of 164 mAhg^−1^ at 1 C after 500 cycles when using the electrolyte consisting of 1 m LiTFSI in triethylene glycol dimethyl ether ((1 m) LiTFSI‐TEGDME). The prepared materials demonstrate competitive stability and capacity comparable to state‐of‐the‐art inorganic cathode materials, combined with superior cost‐effectiveness and sustainability, making them promising candidates for practical applications.

## Graphical abstract

Density functional theory (DFT) guides the design of natural quinones as organic electrode materials (OEMs) for lithium‐ion batteries (LIBs). Potassiation mitigates capacity fading; potassiated 2,6‐dihydroxy‐9,10‐anthraquinone on carbon nanotubes (K_2_(2,6‐DHAQ)@CNTs) retains 164 mAh g^−1^ after 500 cycles at 1 C, using 1 M LiTFSI‐TEGDME as electrolyte. The difference in molecular polarity index (ΔMPI) matches OEMs‐electrolyte pairs.

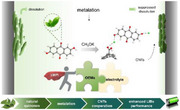

## 1 Introduction

Lithium‐ion batteries (LIBs) are energy storage devices that dominate the market of portable electronics and electric vehicles.^[^ ^1^ ^]^ Commercial LIBs currently depend on the usage of oxides of expensive and often toxic transition metals as cathode materials.^[^ ^2^ ^]^ Organic electrode materials (OEMs) are abundant, potentially of low cost, and environmentally benign, both in terms of raw materials and upon disposal.^[^ ^3^ ^]^ Moreover, their rich chemical space offers a plethora of candidates for high‐performance OEMs for batteries.^[^ ^4^ ^]^ Quinones, belonging to the family of cyclohexanones, have conjugated carbon skeletons and have been extensively studied as OEMs for LIBs because of the high redox activity of the carbonyl groups (C═O) and the fast kinetics during charging/discharging.^[^ ^5^ ^]^ For instance, benzoquinones with perfluoroalkyl groups show a high electrode potential of 3.0 V (vs Li^+^/Li).^[^ ^6^ ^]^ The high abundance in nature^[^ ^7^ ^]^ renders quinone derivatives green and sustainable OEM candidates.^[^ ^8^ ^]^ However, the practical application of quinones is severely restricted by the rapid capacity decay due to their dissolution in the organic electrolyte and their poor electrical conductivity.^[^ ^9^ ^]^

While the latter issue is readily fixed by the incorporation of conducting nanostructures, such as CNTs,^[^ ^10^ ^]^ the former one is less obvious. To suppress the dissolution of quinones, endeavors have been devoted to increasing the electrolyte concentration, including using solid‐state electrolytes.^[^ ^11^ ^]^ Recently, Lu et al. demonstrated a capacity retention of pyrene‐4,5,9,10‐tetraone of 78% after 1000 cycles by using hydrofluoroethers as electrolyte.^[^ ^12^ ^]^ However, very few studies have provided directional design strategies to prevent OEM dissolution.^[^ ^13^ ^]^ The rational development of OEMs would benefit from a convenient and accurate theoretical description of their dissolution trends via an indicator that is easy to calculate while providing sufficient predictive power.^[^ ^14^ ^]^ However, state‐of‐the‐art approaches to describe solubility, e.g., using dissolution Gibbs free energy^[^ ^15^ ^]^ or Hansen solubility parameter,^[^ ^16^ ^]^ demand either costly calculations or require data only accessible via experiments (e.g., the cohesive energy density, melting point, etc.).^[^ ^17^ ^]^ They thus cannot be employed for assessing the solubility of OEM candidates.

**Figure 1:**
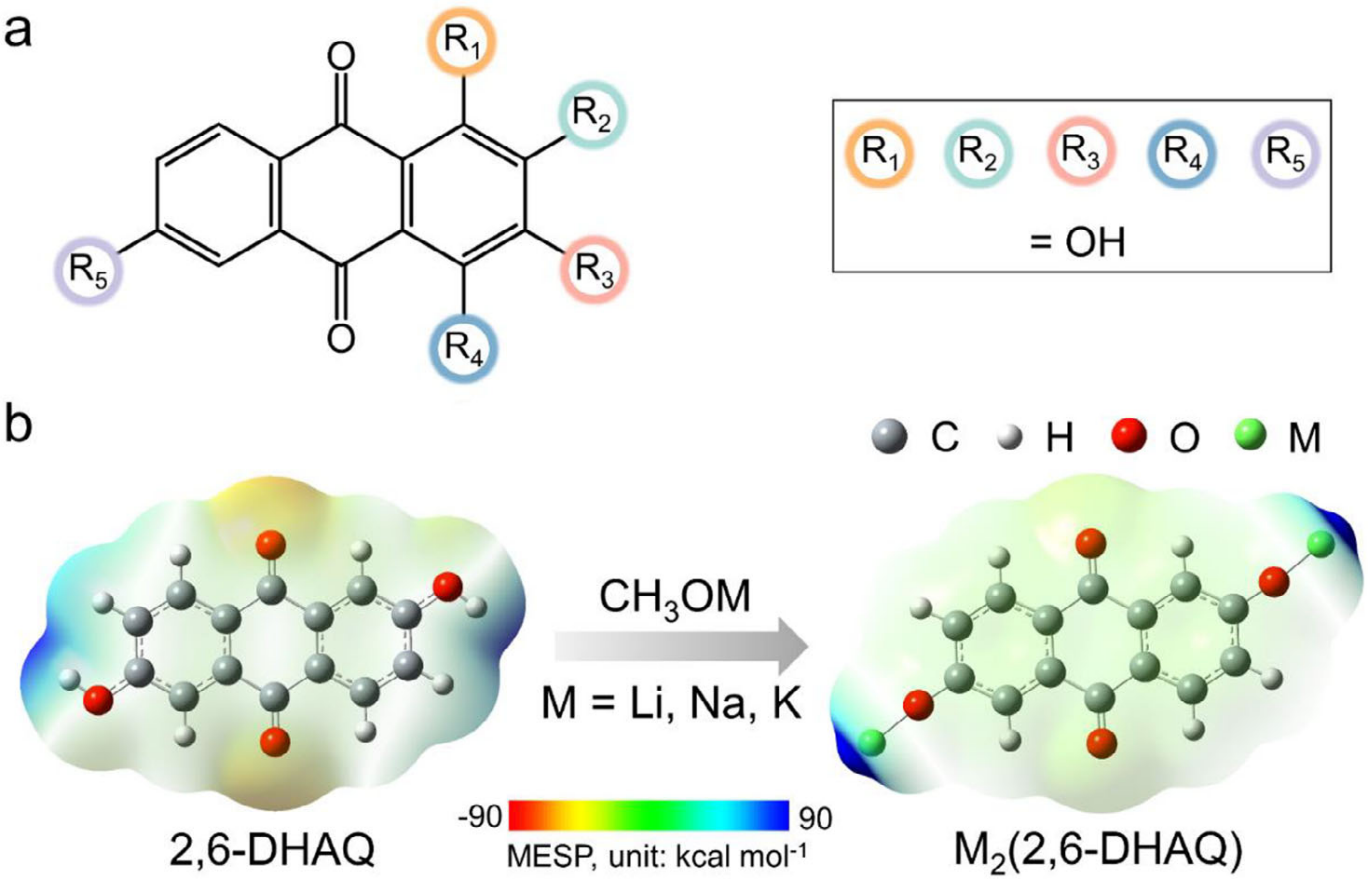
Structure of AQs and their conversion to alkali‐metalated AQ‐salts. a) Structures of AQs that contain hydroxyl groups at indicated positions 1‐5. b) Schematics for the synthesis of alkali‐metalated AQ‐salts from AQs via a simple one‐pot metalation reaction by taking 2,6‐DHAQ as an example. Redistribution of molecular electrostatic potential (MESP) occurs after metalation.

According to the well‐established empirical principle “*like dissolves like*”, OEMs with a similar polarity to that of the electrolyte tend to dissolve easily. Thus, increasing the molecular polarity difference between quinones and the electrolyte should effectively suppress quinone dissolution and thus relieve capacity fading. Natural quinones that contain hydroxyl (─OH) groups (**Figure** **1**) can be easily converted to quinone salts by replacing ─OH with ─OM (M = Li, Na, K). By metalation, the molecular polarity difference between quinones and the electrolyte is increased, which in turn will suppress their dissolution. As metalation also enhances the electric conductivity, it will hence improve the overall electrochemical performance. While ionic bonding was reported to be vital for enhancing the electrochemical performance of carbonyl‐based OEMs for sodium and potassium ion batteries,^[^ ^18^ ^]^ the universal metalation effect on their performance in LIBs and its underlying mechanism remain undisclosed.

Driven by the above considerations, we selected a series of hydroxy‐containing natural anthraquinones (AQs) and investigated the effect of alkali metalation on their electrochemical performance in LIBs. First‐principles calculations were performed to understand the impact of metalation on the redox activity, theoretical capacity, and solubility of AQs with different numbers of ─OH groups at different positions. We propose *the difference in molecular polarity index* (ΔMPI, kcal mol^−1^) to quantify the solubility of AQs and their salts in aprotic electrolytes. We synthesize AQ salts by straightforward one‐pot alkali metalation and subsequently verify their performance in LIB prototypes. We demonstrate both theoretically and experimentally the reliability of metalation in improving the stability of AQs in LIBs, contributing to improved specific capacity and high theoretical capacity attainment (TCA, obtained by calculating the discharge capacities of AQs/AQ‐salts as a percentage of their theoretical capacities). Metalation of AQs with strongly electropositive potassium was found to be the most effective strategy in enhancing the electrochemical performance of AQs. K_2_(2,6‐DHAQ) shows a high‐capacity retention of 87% after 100 cycles when used as a cathode at 0.1 C. After improving the conductivity with the incorporation of CNTs in the electrolyte consisting of 1 m LiTFSI in triethylene glycol dimethyl ether (TEGDME), K_2_(2,6‐DHAQ) shows a high TCA of 97% at 1 C, maintaining the capacity of 164 mAh g^−1^ for 500 cycles.

## 2 Results and Discussion

### 2.1 Computational Screening of AQs‐Salts Concerning their Electronic Structure, Solubility, Redox Activity, and Theoretical Capacity

**Figure 2:**
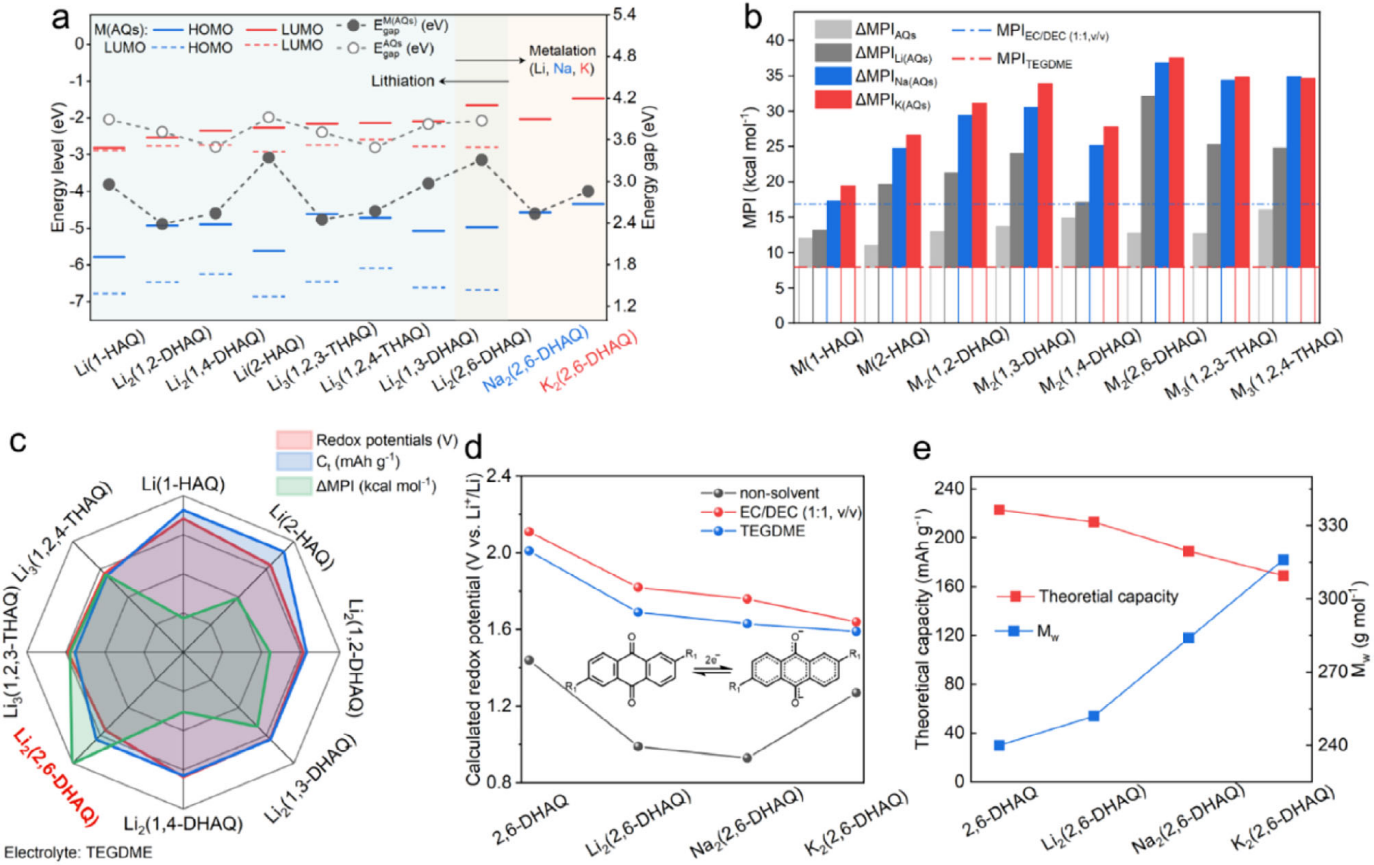
Electronic properties and electrochemical characteristics of AQs and M(AQs). a) The frontier orbital energy levels and HOMO‐LUMO gaps of M(AQs) in comparison with AQs. b) Molecular polarity index (MPI) of AQs/M(AQs) and the electrolyte solvents. The difference of MPI (ΔMPI) values between AQs/M(AQs) and TEGDME is indicated by the solid color bar. c) Radar plots of the electrochemical properties of Li(AQs) when TEGDME is used as the electrolyte solvent. d) Calculated redox potentials in solvent‐free conditions, EC/DEC (1:1, v/v), and TEGDME. e) Theoretical capacities and molecular mass of 2,6‐DHAQ and M_2_(2,6‐DHAQ).

We selected eight natural hydroxy‐/ dihydroxy‐/, and trihydroxyanthraquinone (HAQ/DHAQ/THAQ) isomers,^[^ ^19^ ^]^ i.e., 1‐HAQ, 2‐HAQ, 1,2‐DHAQ, 1,3‐DHAQ, 1,4‐DHAQ, 2,6‐DHAQ, 1,2,3‐THAQ, and 1,2,4‐THAQ (Figure 1; Figure S1, Supporting Information) and their corresponding metalated (M = Li, Na, K) organic salts (M(AQs)), where ─OHs are replaced with ─OMs as target molecules. We first studied the electronic properties of these systems using first‐principles calculations (see Computational Methods, Supporting Information for details). Metalation alters the shape of the highest occupied molecular orbitals (HOMOs), but only slightly affects the lowest unoccupied molecular orbitals (LUMOs) (Figure S2, Supporting Information). It shifts the energy levels of the AQ's frontier orbitals upward (**Figure** **2**a; Figure S3, Supporting Information), whereas the upshift of HOMO is more significant than that of the LUMO, leading to a significant reduction of the HOMO‐LUMO (HL) energy gap from the range of 3.49–3.93 eV for the AQs toward 2.39–3.35, 1.85–2.86, and toward 1.87–2.95 eV of the lithiated, sodiated, and potassiated salts, respectively (Table S1, Supporting Information). The reduced HL gap is correlated with increased molecular aromaticity as indicated by the increased multicenter bond indexes (Table S2 and Figure S4, Supporting Information). The HL gap reduction follows the trend of electropositivity of the metal species. For instance, for 2,6‐DHAQ, it is reduced from 3.87 to 3.31/2.54/2.86 eV for Li/Na/K_2_(2,6‐DHAQ), respectively (Figure 2a). The HL gap reduction is further impacted by the number of hydroxyl groups (more ─OH groups result in stronger HL gap reduction) and by their position (the effect is strongest if ─OH is in the ortho position of the carbonyl group).

A reliable measure of the polarity is the *molecular polarity index* (MPI, kcal mol^−1^, Equation S1, Supporting Information), the surface integral of the MESP.^[^ ^20^ ^]^ Generally, a larger MPI value indicates a higher overall polarity of the molecule. The calculated MPI values of the M(AQ) target molecules and the most common electrolytes are given in Tables S3 and S4 (Supporting Information) and Figure 2b. Inspired by the “*like dissolves like*” principle, we propose the difference of MPI values between solute and solvent (ΔMPI, Equation S2, Supporting Information) as a solubility descriptor. A higher ΔMPI value indicates a larger polarity difference, and thus a more suppressed dissolution. According to Table S5 (Supporting Information), ΔMPI values exceeding 15 kcal mol^−1^ indicate immiscibility, whereas those below 10 kcal mol^−1^ denote solubility, as established by benchmarking against a set of known soluble and insoluble reference compounds. In contrast to computational descriptors that demand extensive DFT and MD calculations to analyze dissolution behavior, ΔMPI affords rapid, scalable assessments of OEM–solvent compatibility, thereby enabling efficient pre‐screening of electrolyte candidates prior to higher‐level theoretical investigations. For our purpose, we thus have to increase the polarity of the M(AQs) and combine it with an electrolyte of low polarity.

Four complementary strategies impact the MPI of the M(AQs):

- Metalation significantly increases the MPI of AQs.
- A higher content of ─OH groups leads to an even more increased MPI after metalation.
- The position of the ─OH group impacts the MPI, where the polarity increase by metalation is higher if the ─OH groups are spatially more separated.
- M(AQs) with more electropositive metals have higher MPI.

Among the most common electrolyte solvents, triethylene glycol dimethyl ether (TEGDME) and EC/DEC (1:1, v/v), the 1:1 volume ratio mixture of ethylene carbonate and dimethyl carbonate, the former one is favored as its MPI is as low as 7.96 kcal mol^−1^ (Table S4, Supporting Information).

Figure 2b summarizes all calculated MPI values of the M(AQs) molecules and compares them with the baseline of the two aforementioned electrolytes. The highest ΔMPI is found between K_2_(2,6‐DHAQ) and TEGDME, which is thus predicted to be the system with the most suppressed OEM dissolution in the electrolyte (Figure S5, Supporting Information). There are some peculiarities evident from Figure 2b. Lithiation of 1‐HAQ and 1,4‐DHAQ results in systems where Li is attracted by two nearby oxygen atoms, and thus in a modest MPI increase. For 1,2,4‐THA, the sodiated salt has a higher MPI than the potassiated one. The polarity of K_3_(1,2,4‐THAQ) is slightly reduced due to the severe deformation of the molecule after potassiation (Figure S6, Supporting Information). We conclude that our computational screening suggests the K_2_(2,6‐DHAQ)/TEGDME system with the highest cycling stability. An additional advantage of TEGDME as an electrolyte is its capability to dissolve the solvent additives LiPF_6_ and LiTFSI.

The redox activity, defined in Equations (S3) and (S4) (Supporting Information), and calculated employing density functional theory (DFT, see Methods, Supporting Information), gives the ability for the carbonyl group to bind with Li. We have first screened the AQs and their lithiated counterparts in the gas phase and in the two electrolyte solvents. Lithiation reduces the redox potential significantly (see Figure S7 and Tables S6 and S7, Supporting Information), which can be rationalized by the upshift of the LUMO of the AQs upon lithiation, and by the reduction of their vertical electron affinities (Figure S8 and Tables S8 and S9, Supporting Information). For example, the calculated redox potential ranges from 2.02 to 2.40 V for AQs and from 1.69 to 2.05 V for their lithiated counterparts in the TEGDME electrolyte. The redox potential in EC/DEC (1:1, v/v) (1.72 to 2.11 V) is slightly higher due to its higher dielectric constant, which agrees well with the result of a previous study.^[^ ^21^ ^]^

In conclusion, our computational screening approach revealed that 2,6‐DHAQ is the most promising AQ (Figure S9, Supporting Information), and its potassiated form in TEGDME electrolyte is expected to be the best‐performing OEM candidate, as it has a comparable redox potential and capacity to other M(AQs) (Figure 2c,d), but a significantly lower solubility due to the large ΔMPI (24.2, 28.9, and 29.6 kcal mol^−1^ for lithiated, sodiated and potassiated salts, respectively, Table S3, Supporting Information). The theoretical capacity of metalated AQs decreases due to the increased molecular weights (Figure 2e). Therefore, their improved stability in the electrolyte is compromised by the slight sacrifice of redox activity and theoretical capacity (Figure S10, Supporting Information).

### 2.2 Experimental Determination of the Solubility and Electrochemical Performance of AQs/M(AQs)

**Figure 3:**
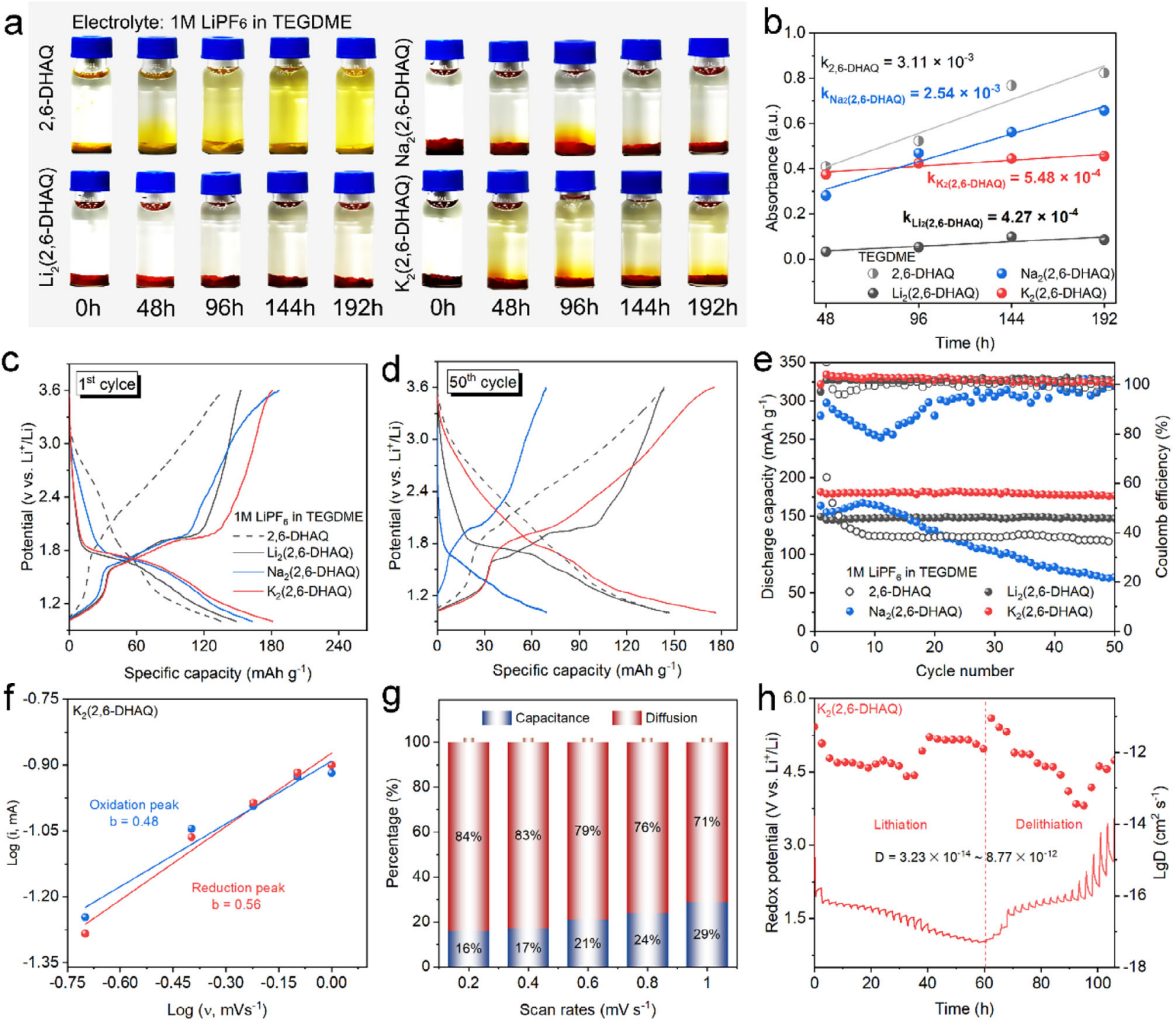
Experimental validation of AQs and (M)AQs as OEMs. a) Photographs of 2,6‐DHAQ and M_2_(2,6‐DHAQ) dissolved in TEGDME‐LiPF_6_ for 48, 96, 144, and 192 h, respectively. b) Highest UV–vis absorption peaks as a function of exposure time and their linear fits for 2,6‐DHAQ and M_2_(2,6‐DHAQ) after soaking in TEGDME‐LiPF_6_ for different hours. The charge–discharge profiles of 2,6‐DHAQ and M_2_(2,6‐DHAQ) in the electrolyte of TEGDME‐LiPF_6_ for the c) 1st and d) 50th cycle, respectively. e) Cycling stability and Coulomb efficiency of 2,6‐DHAQ and M_2_(2,6‐DHAQ) at 0.1 C in TEGDME‐LiPF_6_ with a lifespan of 50 cycles. f) b values of oxidation/reduction peaks of K_2_(2,6‐DHAQ) during cyclic voltammetry measurement at scan rates of 0.2–1.0 mV s^−1^. g) Capacitance and diffusion contribution to the Li^+^ ion intercalation and deintercalation process of K_2_(2,6‐DHAQ) at multiple scan rates. h) GITT curves and the values of D_Li+_ of K_2_(2,6‐DHAQ).

To validate the effectiveness of metalation in reducing the solubility of OEMs in electrolytes, 2,6‐DHAQ and M_2_(2,6‐DHAQ) were synthesized for experimental assessment (Figures S11–S13, Supporting Information). A comprehensive 192‐h solubility measurement of 2,6‐DHAQ and its alkali‐metalated salts was carried out in two common electrolytes (**Figure** **3**a; Figure S14, Supporting Information), i.e., TEGDME and EC/DEC (1:1, v/v) with 1 m LiPF_6_, denoted as LiPF_6_‐TEGDME and LiPF_6_‐EC/DEC (1:1, v/v), respectively. In both cases, the originally colorless solutions become yellow after 2,6‐DHAQ is added for 96 h, demonstrating the dissolution of 2,6‐DHAQ. By contrast, the reddish‐brown M_2_(2,6‐DHAQ) is well maintained in the solution of LiPF_6_‐TEGDME after 192 h, while its dissolution is observed in LiPF_6_‐EC/DEC (1:1, v/v). The higher stability of M_2_(2,6‐DHAQ) in LiPF_6_‐TEGDME is well rationalized by the higher ΔMPI in TEGDME compared to that in EC/DEC (1:1, v/v) (Figure 2b). However, since Na_2_(2,6‐DHAQ) and K_2_(2,6‐DHAQ) possess a more reduced crystal size than that of Li_2_(2,6‐DHAQ) (Figure S13, Supporting Information), the more exposed surface area leads to a relatively more facile dissolution at the beginning. The UV–vis spectra show that the highest absorbance peak exhibits a positive linear correlation with the soaking time in both LiPF_6_‐EC/DEC (1:1, v/v) and LiPF_6_‐TEGDME (Figure 3b; Figures S15 and S16, Supporting Information). The reduced slope of M_2_(2,6‐DHAQ) compared to that of 2,6‐DHAQ in both electrolytes unambiguously demonstrates the effectiveness of metalation for suppressing dissolution of 2,6‐DHAQ. The thermal stability of 2,6‐DHAQ is also significantly improved after metalation. As indicated by Figure S17 (Supporting Information), 2,6‐DHAQ is decomposed at 400 °C in N_2_ atmosphere, but remains stable up to 600 °C after metalation. The low dissolution and high thermal stability of M_2_(2,6‐DHAQ) make them ideal candidates for application in LIBs.

The electrochemical performance of 2,6‐DHAQ and M_2_(2,6‐DHAQ) in both LiPF_6_‐TEGDME and LiPF_6_‐EC/DEC (1:1, v/v) was ultimately investigated experimentally in LIBs (see setup details in the Methods Section, Supporting Information). The lower cutoff voltage was fixed at 1.0 V versus Li^+^/Li to remain consistent with the protocols established for quinone‐based organic cathode materials.^[^ ^22^ ^]^ This threshold is widely accepted as a practical boundary that suppresses parasitic reactions such as electrolyte decomposition or lithium‐dendrite formation while retaining the dominant redox activity of the quinone core. While lowering the cutoff below 1.0 V versus Li^+^/Li may uncover additional capacity, it would simultaneously increase the risk of these side reactions and compromise long‐term cyclability. The discharge and charge profiles for the 1st and 50th cycles in LiPF_6_‐TEGDME are shown in Figure 3c,d, respectively. M_2_(2,6‐DHAQ) shows a more plateau‐shaped voltage profile than 2,6‐DHAQ, indicating a more stable voltage output after metalation. In agreement with our theoretical predictions, the discharge voltage decreases from 2.03 to 1.67/1.70/1.70 V after lithiation/sodiation/patassiation, resulting from the reduced electron affinity of the AQs after metalation. The cycling stability of 2,6‐DHAQ is significantly improved after lithiation and potassiation in LiPF_6_‐TEGDME, which is as predicted earlier, better than that in LiPF_6_‐EC/DEC (1:1, v/v) (Figures S18 and S19, Supporting Information) because of the more suppressed dissolution. However, for Na_2_(2,6‐DHAQ), the specific capacity degrades dramatically after 15 cycles, which results from its anomalous energy storage mechanism (Figure S20, Supporting Information). By performing systematic cyclic voltammetry (CV) measurements at different scan rates, it has been observed that Na_2_(2,6‐DHAQ) undergoes a capacitance process during charging and discharging, similar to that of the original 2,6‐DHAQ (Figure S21, Supporting Information), but with an even greater dominance of the capacitive behavior (Figure S20, Supporting Information). By contrast, Li_2_/K_2_(2,6‐DHAQ) proceeds through a Faradaic charge–discharge process (Figure 3f,g; Figures S22 and S23, Supporting Information). There is a linear relation between the logarithm of the peak current (log i) and that of the scan rate (log ν),^[^ ^23^ ^]^ with a reduction slope of 0.83 and 0.78 for 2,6‐DHAQ and Na_2_(2,6‐DHAQ) (Figures S21a and S20a, Supporting Information), respectively, confirming a capacitive‐controlled process, while the slopes of 0.49 and 0.56 for Li_2_(2,6‐DHAQ) and K_2_(2,6‐DHAQ) (Figure S22a, Supporting Information; Figure 3f), respectively, indicate a diffusion‐limited one (Figure 3g).^[^ ^24^ ^]^ Note that the b value for the reduction peak of Li_2_(2,6‐DHAQ) is only marginally below the theoretical limit of 0.5 and within typical experimental uncertainty, especially given the noise common in CV measurements at low scan rates. For the oxidation peaks, slightly lower *b*‐values were observed for Li_2_(2,6‐DHAQ) (0.43) and K_2_(2,6‐DHAQ) (0.48), which can be additionally ascribed to the polarization at low current densities (Figure S24, Supporting Information), destabilizing the electrode–electrolyte interface and hindering reaction kinetics.

K_2_(2,6‐DHAQ) shows better cycling performances (Figure 3e) than all the other examined candidates, while possessing comparable redox activity and voltage output as Li_2_(2,6‐DHAQ). It maintains a discharge capacity of 176 mAh g^−1^ at 0.1 C after 50 cycles, much higher than that of Li_2_(2,6‐DHAQ) (147 mAh g^−1^) and 2,6‐DHAQ (117 mAh g^−1^). Remarkably, the discharge capacity retention of K_2_(2,6‐DHAQ) is higher than 97% after 50 cycles, demonstrating that the cycling stability of 2,6‐DHAQ is significantly improved after potassiation. The enhanced Li‐ion diffusion coefficient (D_Li+_) and reduced electrical resistance of 2,6‐DHAQ after potassiation (Figure 3h; Figures S25 and S26, Supporting Information) improve its electrochemical performance, as confirmed by CV, galvanostatic intermittent titration technique (GITT) (Figure S25, Supporting Information), and electrochemical impedance spectroscopy (EIS) (Figure S26, Supporting Information). The D_Li+_ value of Li_2_(2,6‐DHAQ), Na_2_(2,6‐DHAQ), and K_2_(2,6‐DHAQ) are in the range 9.58 × 10^−15^–1.46 × 10^−11^, 9.41 × 10^−14^–6.42 × 10^−12^, and 3.23 × 10^−14^–8.77 × 10^−12^, respectively, higher than that of 2,6‐DHAQ (6.77 × 10^−15^–4.77 × 10^−12^), revealing a faster Li^+^ mobility during the lithiation/delithiation process after metalation. Furthermore, the charge transfer resistance of K_2_(2,6‐DHAQ) observed in Figure S26 (Supporting Information) is ≈6 times smaller than that of pristine 2,6‐DHAQ, demonstrating that the conductivity of AQs can be effectively improved after metalation. However, will ion exchange jeopardize the durability of the LIBs? Electrochemical measurements combined with in situ XRD measurements^[^ ^25^ ^]^ illustrated in Figures 3c–e and S27 (Supporting Information) show no ion exchange between K_2_(2,6‐DHAQ) and LiPF_6_‐TEGDME electrolyte during charging and discharging. The stability of K_2_(2,6‐DHAQ) is demonstrated by the electrochemical performance (Figure 3c–e), which remains stable over 50 cycles, with no features indicating cation exchange. The absence of peak shifts or intensity changes in the in situ XRD patterns (Figure S27, Supporting Information) aligns with the charge storage mechanism typical of organic small‐molecule electrodes like 2,6‐DHAQ, which undergoes reversible redox reactions at the carbonyl (C═O) groups, preserving its molecular framework instead of inducing solid‐phase transformation. Thus, the above results validate the effectiveness of potassiation in suppressing the dissolution of OEMs in aprotic electrolytes, contributing to significantly improved cycling stability. Although the voltage of K_2_(2,6‐DHAQ) (1.70 V vs Li^+^/Li) is lower than that of many inorganic cathodes. This work highlights a sustainable and cost‐effective alternative, particularly suitable for applications where energy density is secondary to environmental impact and material sustainability—such as large‐scale grid storage. This finding is in perfect alignment with the theoretical predictions previously established.

**Figure 4:**
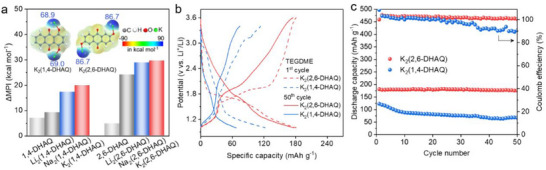
The structure‐property relationship of AQ salts with different positions of side chain functional groups and their electrochemical performance. a) The ΔMPI values of 1,4‐DHAQ and 2,6‐DHAQ in TEGDME before and after metalation. Inside are geometries and MESP of K_2_(1,4DHAQ) (left) and K_2_(2,6‐DHAQ) (right). b) Charge–discharge profiles of K_2_(2,6‐DHAQ) and K_2_(1,4‐DHAQ) in the electrolyte of TEGDME for the 1st and 50th cycle at 0.1 C. c) Cycling performance and Coulomb efficiency of K_2_(2,6‐DHAQ) and K_2_(1,4‐DHAQ) at 0.1 C, with a lifespan of 50 cycles.

Our DFT calculations indicate that the properties of AQ isomers with the ─OH groups at different positions vary in their properties before and after metalation (Figure 2). Therefore, we examined experimentally their electrochemical performance (**Figure** **4**a; Figure S7, Supporting Information). Among the two most abundant isomers, K_2_(1,4‐DHAQ) and K_2_(2,6‐DHAQ), the former one has a slightly higher initial discharge potential (Figure 4b and **Table** **1**). However, it shows a steeper potential as a function of the specific capacity, a lower discharging capacity after 50 cycles, and hence a lower stability compared to K_2_(2,6‐DHAQ). The superior performance of K_2_(2,6‐DHAQ) is understood by its enhanced polarity, resulting in suppressed dissolution in TEGDME (Table S10 and Figure S28, Supporting Information), as anticipated by the larger ΔMPI values (Figure 4a). A further benefit of the good electrochemical performance of K_2_(2,6‐DHAQ) is its diffusion‐dominated charging and discharging mechanism, compared to the capacitive‐dominated one for K_2_(1,4‐DHAQ) (Figure S29, Supporting Information). The capacity retention of K_2_(2,6‐DHAQ) is remarkably 97% after 50 cycles, much higher than that of isomeric K_2_(1,4‐DHAQ).

**Table 1:** Comparison of the theoretical and experimental electrochemical performance (a lifespan of 50 cycles at 0.1 C) of 2,6‐DHA, M_2_(2,6‐DHAQ), and K_2_(1,4‐DHAQ), including theoretical and experimental redox potential (E), capacity (C), ΔMPI, the Hildebrand solubility parameter differences (Δδ), and capacity retention.

Performance	Structures				
	2,6‐DHAQ	Li_2_[2,6‐DHAQ]	Na_2_[2,6‐DHAQ]	K_2_[2,6‐DHAQ]	K_2_[1,4‐DHAQ]
E_theo_ (V)	2.02	1.69	1.63	1.59	2.09
E_exp_ (V)	2.03	1.67	1.70	1.70	1.77
C_theo_ (mAh g^−1^)	223	213	189	169	169
C_exp_ (mAh g^−1^)	117	147	70	176	67
ΔMPI (kcal mol^−1^)	4.8	24.2	28.9	29.6	19.8
Δδ (MPa^0.5^)	4.5	11.4	13.7	13.2	5.2
Retention	59%	99%	43%	97%	54%

**Figure 5:**
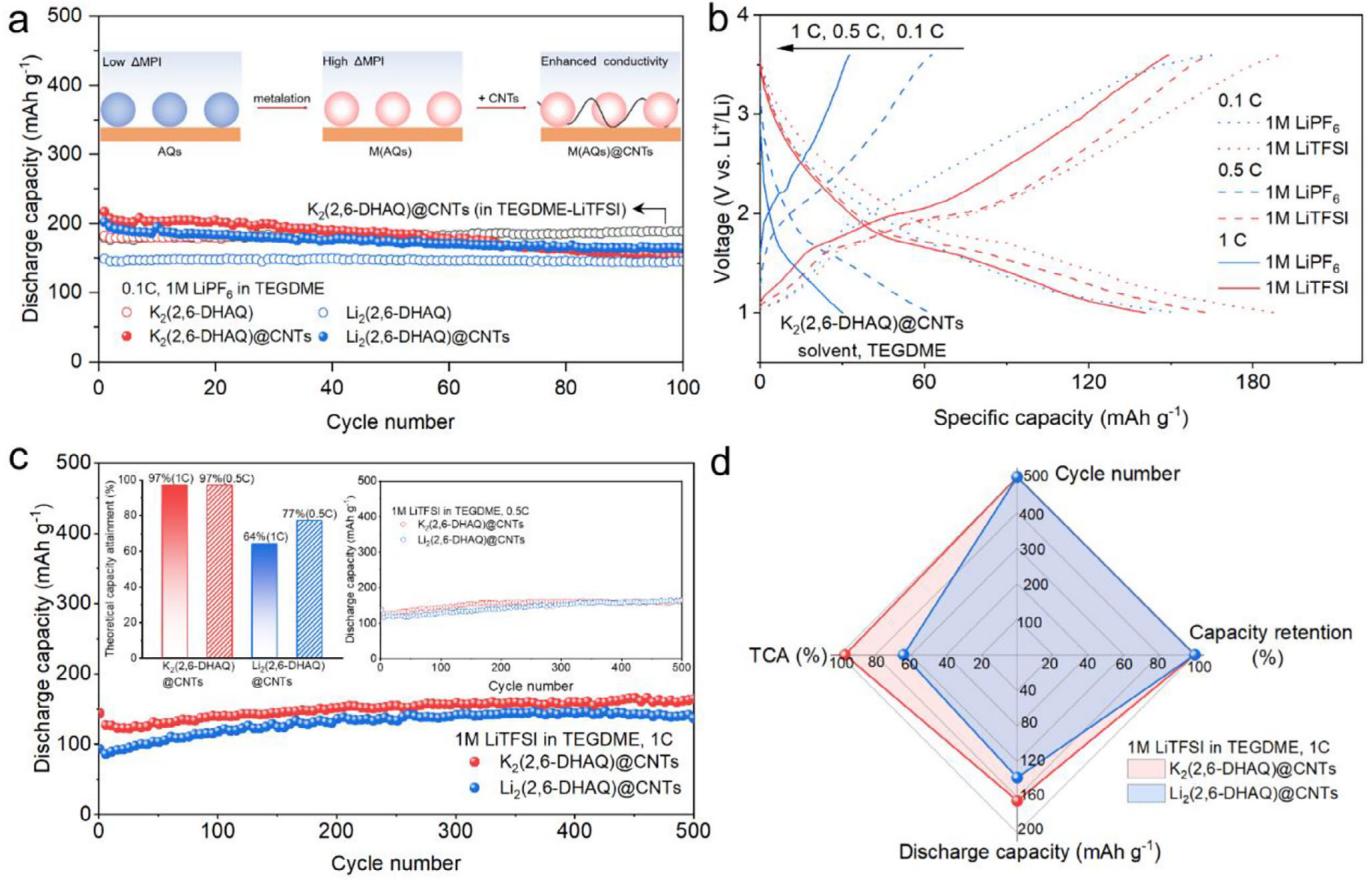
Electrochemical performance of K/Li_2_(2,6‐DHAQ)@CNTs. a) Cycling performance of K/Li_2_(2,6‐DHAQ) and K/Li_2_(2,6‐DHAQ)@CNTs at 0.1 C, with a lifespan of 100 cycles. Inset: schematics for the improved stability of AQs achieved by metalation and incorporation of CNTs. b) Charge–discharge profiles of K_2_(2,6‐DHAQ)@CNTs at 0.1 C, 0.2 C, and 0.5 C after 100 cycles in the electrolyte of TEGDME with 1 m LiPF_6_ and LiTFSI, respectively. c) Cycling stability of K/Li_2_(2,6‐DHAQ)@CNTs at 1 C, with a life of 500 cycles. Inset: TCA (1C, left) and cycling stability (0.5 C, right) of K/Li_2_(2,6‐DHAQ) @CNTs. d) Radar plots of electrochemical performance of K/Li_2_(2,6‐DHAQ) @CNTs at 1 C, in TEGDME‐LiTFSI electrolyte.

Although metalation improves the conductivity of metalated AQs, it is still below that of the state‐of‐the‐art inorganic electrode materials for high‐rate LIBs (Figure S26, Supporting Information). A limiting factor is the poor conductivity of OEMs, which also affects their cycling stability. The conductivity can, according to previous studies, be enhanced by the incorporation of carbon nanotubes (CNTs).^[^ ^26^ ^]^ Therefore, we added CNTs modified with carboxyl functional groups (─COOH) to K/Li_2_(2,6‐DHAQ) and studied the performance of the composite electrode material (**Figure** **5**a). We observed an enhanced discharge capacity, especially for K_2_/Li_2_(2,6‐DHAQ) at 0.1 C in LiPF_6_‐TEGDME, which we rationalize by the enhanced conductivity and reduced particle size after the incorporation of CNTs (Figure S30, Supporting Information). The carboxylate groups further stabilize K_2_/Li_2_(2,6‐DHAQ) in a unique interfacial synergy by forming ionic bonds with the metal atoms. After potassiation and CNT modification, the resistance of the cathode is reduced to 116.4 Ω (Figure S31, Supporting Information). This value is 73% and 58% lower than that of potassium salt (K_2_(2,6‐DHAQ)) and CNT‐modified precursor (2,6‐DHAQ@CNT/COOH), respectively. The relatively high CNT content employed in this work addresses the inherent challenges of small‐molecule organic electrodes, e.g., the poor electronic conductivity and insufficient interparticle contact—particularly under cycling conditions that promote dissolution. As benchmarked in Table S11 (Supporting Information), such conductive additive loadings represent a common requirement in the field to ensure efficient charge transport and enhanced electrode robustness. While this design unavoidably introduces sloped voltage profiles and pseudocapacitive contributions, it constitutes a critical trade‐off to achieve viable reaction kinetics and sustained cyclability for practical implementation.

Interestingly, K_2_/Li_2_(2,6‐DHAQ) shows improved electrochemical performance when replacing LiPF_6_ with LiTFSI in the electrolyte. In specific, the capacity of K_2_/Li_2_(2,6‐DHAQ)@CNTs remains at 189/176 mAh g^−1^ after 100 cycles at 0.1 C in TEGDME‐LiTFSI, and the rate performance is improved (Figure 5b; Figure S32, Supporting Information). The higher capacity and more stable voltage output of K_2_/Li_2_(2,6‐DHAQ)@CNTs in LiTFSI‐TEGDME is ascribed to its higher stability than that in LiPF_6_‐TEGDME, as illustrated by UV–vis absorption measurement (Figure S33, Supporting Information) and higher interfacial stability as demonstrated by the dynamic EIS measurements (Figure S34, Supporting Information). As a result, K_2_/Li_2_(2,6‐DHAQ)@CNTs exhibit a high discharge capacity of 164 and 137 mAh g^−1^, respectively, after 500 cycles at 1 C (Figure 5c). The TCA, discharge capacity, capacity retention, and redox potential of K_2_/Li_2_(2,6‐DHAQ)@CNTs are 97/64%, 164/137 mAh g^−1^, 100%/100%, respectively. We demonstrate that the addition of CNTs, along with the appropriate utilization of electrolyte solute (LiTFSI), further improves the stability of metalated AQs in traditional aprotic electrolytes, leading to high‐capacity retention over extended cycling and improved high‐rate performance in LIBs. Note that although the redox potential of K_2_(2,6‐DHAQ)@CNTs (1.71 V vs Li^+^/Li) and its resulting energy density (280 Wh kg^−1^) are modest relative to those of traditional inorganic cathodes, this trade‐off is worthwhile to prioritize sustainability, cost‐effectiveness, and environmental friendliness, making K_2_(2,6‐DHAQ)@CNTs particularly well‐suited for stationary energy storage applications (such as grid storage) where long‐term stability, resource availability, and eco‐compatibility outweigh the need for high energy density.

## 3 Conclusion

Our combined theoretical and experimental investigation demonstrates that metalation is a straightforward, effective, and universal strategy to improve the electrochemical performance of AQs by suppressing their dissolution in commonly used electrolytes. The predicted trend given by our ΔMPI calculations agrees well with that described by the Hildebrand solubility parameter differences (Δδ) (Table 1; see more details in Equation S7, Supporting Information). Therefore, ΔMPI is a dependable, easy‐to‐calculate indicator to indirectly describe the solubility of AQs in electrolytes, which can be generalized to develop effective strategies for enhancing the stability of OEMs and to select appropriate electrolytes for specific OEMs.

According to our results, the electrochemical performance of OEMs is determined by a variety of factors, including not only the redox activity, theoretical capacity, solubility, conductivity, and morphology of the materials themselves but also the selection of electrolytes. The design and development of new OEMs should comprehensively consider and balance these factors. Here, alkali metalation was found to be an effective way to prevent dissolution and thus to enhance the cycling stability of natural quinones, without compromising the merits of these highly abundant materials.

The cycling stability and TCA of K_2_(2,6‐DHAQ)@CNTs demonstrated in this study surpass that of previously reported AQ‐based cathodes (Table S11, Supporting Information) for LIBs, demonstrating that metalation is a straightforward but effective strategy to improve the electrochemical performance of OEMs. While the above results consolidate the merits of metalation, this work also advances the field in three specific directions that have received limited attention. First, we deliberately limited chemical modification to natural hydroxy‐quinones, underscoring a low‐cost, scalable route to green OEMs. Second, we introduced ΔMPI as a rapid, experiment‐friendly complement to computationally intensive solubility metrics, facilitating high‐throughput screening of electrolyte‐electrode pairs. Third, we uncovered a 58% reduction in interfacial resistance when K^+^ coordination is coupled with CNT networks, highlighting a synergistic design principle for hybrid organic electrodes. The specific capacity of K_2_(2,6‐DHAQ)@CNTs is ≈160 mAh g^−1^, which is also comparable to that of the commercialized cathode material (LiMn_2_O_4_, ≈120 mAh g^−1^), rendering it a green OEM candidate for real practice. These insights, together with the demonstrated performance, position metalated natural quinones as promising, sustainable cathode materials for next‐generation energy storage. Our investigation opens up a new avenue to utilize organic molecules derived from biomass as environmentally‐friendly materials for sustainable energy storage devices.

## Conflict of Interest

The authors declare no conflict of interest.

## Supporting information



Supporting Information

## Data Availability

All data needed to evaluate the conclusions in the paper are present in the paper and/or the Supporting Information. The data that support the findings of this study are openly available in NOMAD at https://nomad‐lab.eu/prod/v1/gui/user/uploads/upload/id/vM5AmUTYRSGyM‐IzqfXR4Q. Structure information of alkali‐metalated anthraquinone salts, and hydroxyl‐substituted natural anthraquinone derivatives can be found in NOMAD with DOI 10.17172/NOMAD/2024.03.25‐1 and DOI 10.17172/NOMAD/2024.03.25‐2, respectively.
