# Supplementary material for: Alkali Metalation Enables Natural Anthraquinone Derivatives as Sustainable Cathode Materials for Lithium‐Ion Batteries
Source: Adv Sci (Weinh). 2025 Aug 30;12(44):e13052. doi: 10.1002/advs.202513052 (PMC12667472; doi:10.1002/advs.202513052)
Supplement: Supplementary file 1 — Supporting Information [file ADVS-12-e13052-s001.pdf]

Supporting Information

Alkali Metalation Enables Natural Anthraquinone Derivatives as Sustainable Cathode Materials for Lithium-ion Batteries

Xinyue Zhu,[a] Xianlong Zhou,[a] Lingchao Cai,[a] Thomas Heine,[b,c,d] * Yu Jing[a]*

[a] Jiangsu Co-Innovation Centre of Efficient Processing and Utilization of Forest Resources, College of Chemical Engineering, Nanjing Forestry University, Nanjing 210037, China

[b] TU Dresden, Fakultät für Chemie und Lebensmittelchemie, Bergstraße 66c, 01062 Dresden, Germany

[c] Center for Advanced Systems Unerstanding (CASUS), Helmholtz-Zentrum Dresden-Rossendorf, Am Untermarkt 2, 02826 Görlitz, Germany

[d] Department of Chemistry and ibs-cnm, Yonsei University, Seodaemun-gu, Seoul 120-749, Republic of Korea

To whom correspondence should be addressed. Email: thomas.heine@tu-dresden.de (TH) and yujing@njfu.edu.cn(YJ)

Computational Section

DFT calculations

Gaussian 16[1] and Multiwfn_3.8[2] packages were employed to perform DFT calculations and to illustrate the structural and electronic properties of eight quinone derivatives and their salts, respectively. The London-dispersion corrected B3LYP-D3(BJ)[3,4] functional with the standard basis set 6-311G (d,p) was used to optimize the structures. The vibrational frequencies of the molecules were computed at the same level. The B2PLYP-D3[5] functional with def2tzvp basis set was used to substantiate the energies of the systems. The solvation free energy was obtained by using a universal solvation model (SMD) together with the M052X/6-31g(d) functional, as recommended elsewhere.[6] Here, we used literature values for the static dielectric constants, i.e. ε = 7.78 for TEGDME[7] and ε = 49.06 for EC/DEC (1:1, v/v).[8] See Supplementary Information for more computational details.

Computational Details

The molecular polarity index (MPI, kcal mol^{-1})[9] is described by the following equation:

$$MPI = (1/A) \iint_S |V(r)| dS \qquad (1)$$

Where V is the molecular electrostatic potential and A is the molecular surface area. The larger the MPI value, the greater is the overall polarity. In addition, similar to calculating the dielectric constant of electrolyte mixtures[10], the MPI value of a mixed solvent is defined as a volumetrically weighted average of the MPI values of its individual components. Then the index (ΔMPI) to quantify the miscibility between AQs and electrolyte solvent molecules was given as follows:

$$\Delta MPI = |MPI_{OEM} - MPI_{solvent}| \qquad (2)$$

Where MPI_{OEM} and $MPI_{solvent}$ present the molecular polarity index of the investigated organic molecule and the electrolyte solvent molecule, respectively.

The thermodynamic parameter, namely, changes in the Gibbs free energies of molecules during redox reactions in presence of solvent were used to determine the redox activity, which is defined by the following equation:

$$\Delta G_{sol} = G^{sol}_{OEM\text{-}nM} - G^{sol}_{OEM\text{-}(n\text{-}1)M} - G^{sol}_{M} \qquad (3)$$

Where $G^{sol}_{OEM\text{-}nM}$, $G^{sol}_{OEM\text{-}(n\text{-}1)M}$ (n=1,2,3) and G^{sol}_{M} denote Gibbs free energies of OEM-nM, OEM-(n−1)M and Li atom in solution at 298.15 K, respectively.

The redox potential (V) was calculated according to equation:

$$V = -\Delta G_{sol}/nF \qquad (4)$$

Here, n is the number of transferred electrons, and F indicates the Faraday constant (F = 96485.3329 sA mol^{-1}).

The theoretical capacity (C_t) was calculated by the following equation:

$$C_t = nF/3.6M_w \qquad (5)$$

Where M_w represents the molecular weight of AQs and AQ-salts investigated in this work.

The redox potential of target molecules was interpreted by calculating the vertical electron affinity (VEA), which is defined by:

$$VEA = E(N) - E(N+1) \qquad (6)$$

Where E(N) and E(N+1) indicate energies of the molecule which is electrically neutral and gains an electron, respectively.

Molecular dynamics (MD) Simulations

Hildebrand solubility parameter (δ, $MPa^{0.5}$) was used to describe the solubility and validate the ΔMPI predictions, which is defined as follows:

$$\delta = (\frac{\Delta E_v}{V_m})^{1/2} = (\frac{\Delta H_v - RT}{V_m})^{1/2} \qquad (7)$$

Where δ indicates the square root of cohesive energy density which is defined by Hildebrand et al[11,12], ΔE_v is change in internal energy of vaporization, and V_m is molar volume. Two materials are thermodynamically miscible when they have similar δ values.

Molecular dynamics (MD) method was performed using the GROMACS 2018.8 package[13] to calculate corresponding parameters to obtain δ. The molecular structures of TEGDME, 2,6-DHAQ, M_2(2,6-DHAQ) and K_2(1,4-DHAQ) were constructed and optimized through Gaussian 16, and these initial structures for MD calculations were built by Packmol software[14]. The dimensions of initial cubic cells were 5 × 5 × 5nm, with periodic boundary conditions. 300 molecules contained in each cell were then generated. In addition, topology files were generated by Sobtop package[15]. After completing energy minimization and pre-equilibrium calculations, production simulations were carried out. Structures were then subjected to MD simulations using the NVT ensemble for at least 2 ns with a step size of 0.001 ps. Cohesive energy density (CED) of these compounds was computed from the last 200 ps of data.

The Hildebrand solubility parameter differences (Δδ) were calculated to describe the miscibility of 2,6-DHAQ, M_2(2,6-DHAQ), and K_2(1,4-DHAQ) in TEGDME.[16] A larger Δδ value indicates a lower miscibility of OEMs with the electrolyte. As shown in Table 1, the ΔMPI predictions agree well with the Δδ predicted dissolution trends, which thus are dependable and save the cost for molecular dynamics simulations (to obtain Δδ). Therefore, ΔMPI is a dependable indicator to indirectly describe the solubility of AQs and can be easily used to primarily predict the dissolution tendency and stability of OEMs.

Experimental Section

Materials

2,6-Dihydroxyanthraquinone (2,6-DHAQ, 97%), lithium methoxide ($LiOCH_3$, 10 wt% in methanol), sodium methoxide ($NaOCH_3$, 99%), potassium methoxide ($KOCH_3$, 98.5%), and methanol (≥ 99.9%) were purchased from Energy Chemical Company. All chemicals were used as received without further purification.

Synthesis of M_2(2,6-DHAQ)

M_2(2,6-DHAQ) (M = Li, Na, K) were synthesized by one-pot solution method**Fehler! Textmarke nicht definiert.**. 5 mmol 2,6-DHAQ was first dissolved in 140 mL of acetone with intense agitation and ultrasonic dispersion. Then 10 mmol $MOCH_3$ was added into 10 mL MeOH. After stirring thoroughly, the acetone solution was added drop by drop to the methanol solution to get them mixed. After 5 hours of condensation reflux, the fully reacted mixed solution is evaporated to remove the solvent through distillation to obtain the primary product. The product was washed with acetone for more than three times, the product was dried at 80 ℃ for 24 hours in a vacuum oven. In order to further enhance the electrochemical performance of active materials, freeze-drying method was employed to decrease the particle size.

Material characterization

The morphology of prepared M_2(2,6-DHAQ) sample was characterized using field-emission scanning electron microscopy (SEM, Regulus 8100, Japan). X-ray diffraction (XRD Ultima IV, Japan) patterns of M_2(2,6-DHAQ) were collected from range of 5 – 80° at a rate of 10°/min. Fourier transform infrared spectrometer (FT-IR) was utilized to detect molecular vibrational information in the wavelength range of 400 – 4000 cm^{-1}. Thermalgravimetric (TGA 209 F3, Germany) analysis was carried out by operating TG 209 F3 Tarsus from room temperature to 800 ℃ at a heating rate of 5 ℃ min^{-1} under N_2 atmosphere. UV-vis spectra (Shimadzu, Japan) were depicted at the range of 200 - 800 nm.

Electrochemical measurements

All electrochemical tests were conducted in two-electrode system at room temperature using electrochemical workstation (DH7002A, Donghua Analytical Instrument, China) and battery testing system (NEWARE). The 2032 coin-type cell was fabricated in a glovebox filled with pure Ar (APURIS, China) by assembling coin cell cap, wave spring, spacer, a lithium foil, a Celgard 2325 separator, AQ/AQ-salt cathode, and coin cell can. Specifically, a commonly used EC/DEC (1:1, v/v) mixture has been initially employed as the electrolyte solvent. The liquid electrolyte (1M $LiPF_6$ in EC/DEC (1:1, v/v), LB-008, 1M $LiPF_6$ in TEGDME, LB-120, and 1M LiTFSI in TEGDME, LK-001) were purchased from DoDoChem. The working electrodes were fabricated by casting a mixture of M_2(2,6-DHAQ), Super P, and PVDF (3: 6 :1 by weight) on Al foil, and a mixture of K/Li_2(2,6-DHAQ), carbonyl CNTs, Super P, and PVDF (3 : 3 : 3 : 1 by weight) on Al foil, which was then dried in vacuum at 80 ℃ for 24 hours. The cyclic voltammetry analysis and galvanostatic charge-discharge tests were performed in the voltage window of 1.0 – 3.6 V. The electrochemical impedance spectroscopy (EIS) of 2,6-DHAQ and M_2(2,6-DHAQ) was tested over a frequency range of 10^{-2} - 10^5 Hz. The galvanostatic intermittent titration technique (GITT) is used to evaluate Li^+ diffusion coefficients in half-cell systems.

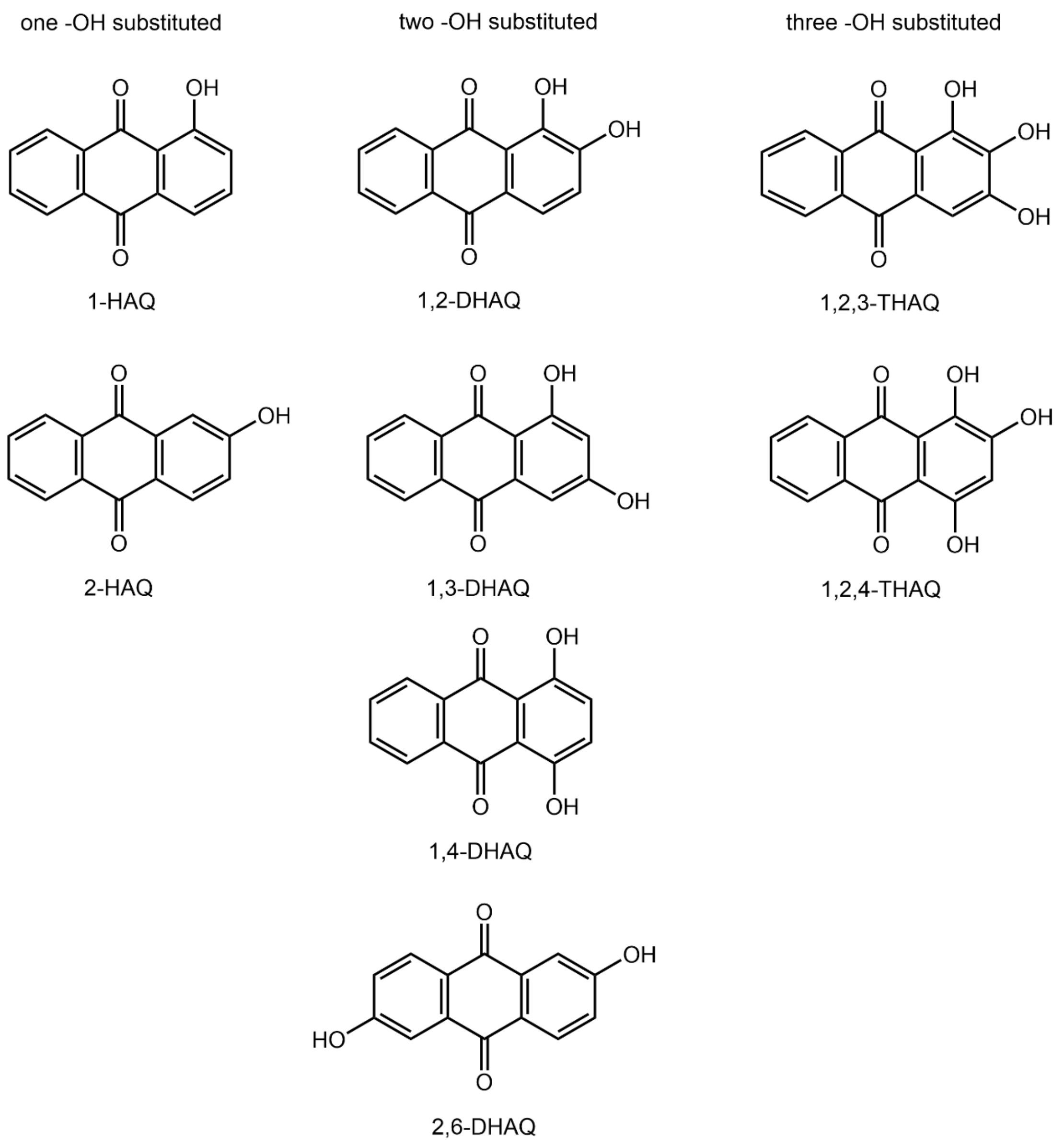

Figure S1. Molecular structures.

Structures of eight natural anthraquinone derivatives (AQs) containing different numbers of hydroxyl groups at different positions.

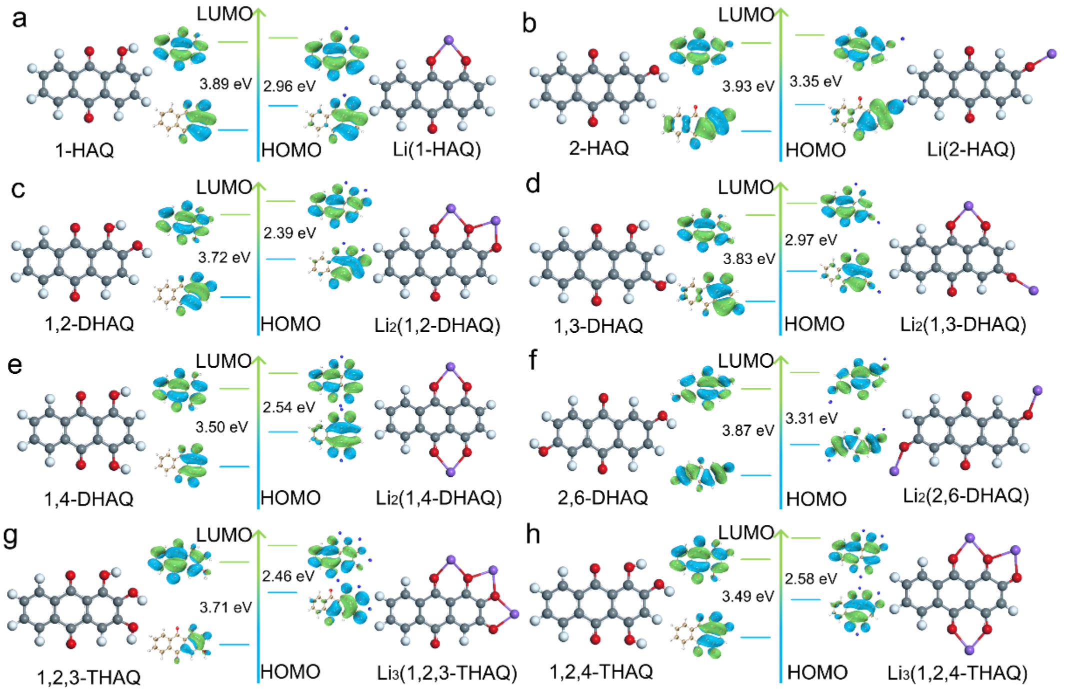

Figure S2. Geometric and electronic structures of natural AQs and their corresponding lithium salts. Structure and frontier orbitals of (a) 1-HAQ/Li(1-HAQ), (b) 2-HAQ/Li(2-HAQ), (c) 1,2-DHAQ/Li_2(1,2-DHAQ), (d) 1,3-DHAQ/Li_2(1,3-DHAQ), (e) 1,4-DHAQ/Li_2(1,4-DHAQ), (f) 2,6-DHAQ/Li_2(2,6-DHAQ), (g) 1,2,3-THA/Li_3(1,2,3-THAQ), and (h) 1,2,4-THAQ/Li_3(1,2,4-THAQ).

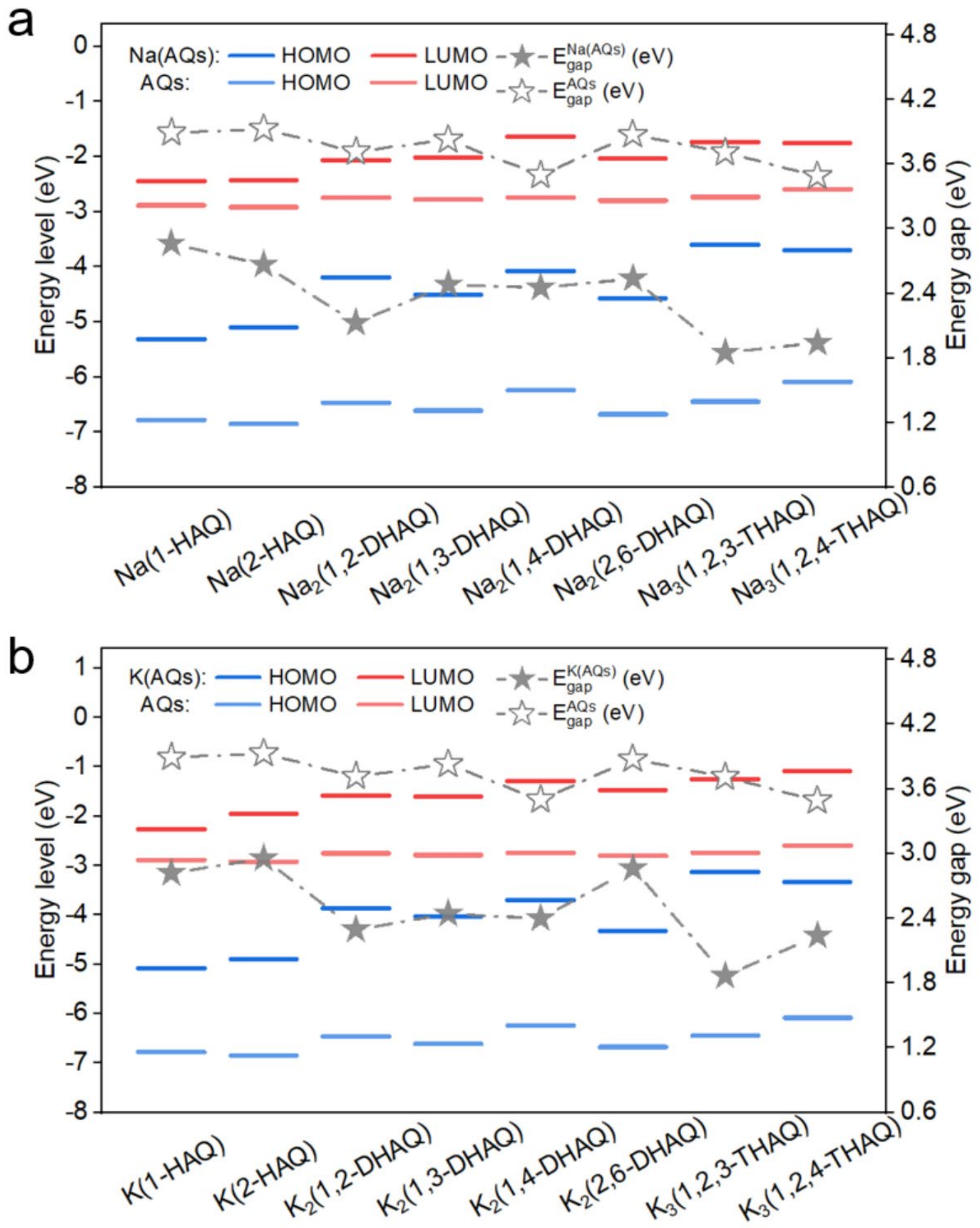

Figure S3. Electronic properties of Na(AQs) and K(AQs). (a) Energy level (eV) for frontier orbitals and HOMO-LUMO gaps (eV) of sodiated AQs and (b) potassiated AQs in comparison with that of the original AQs.

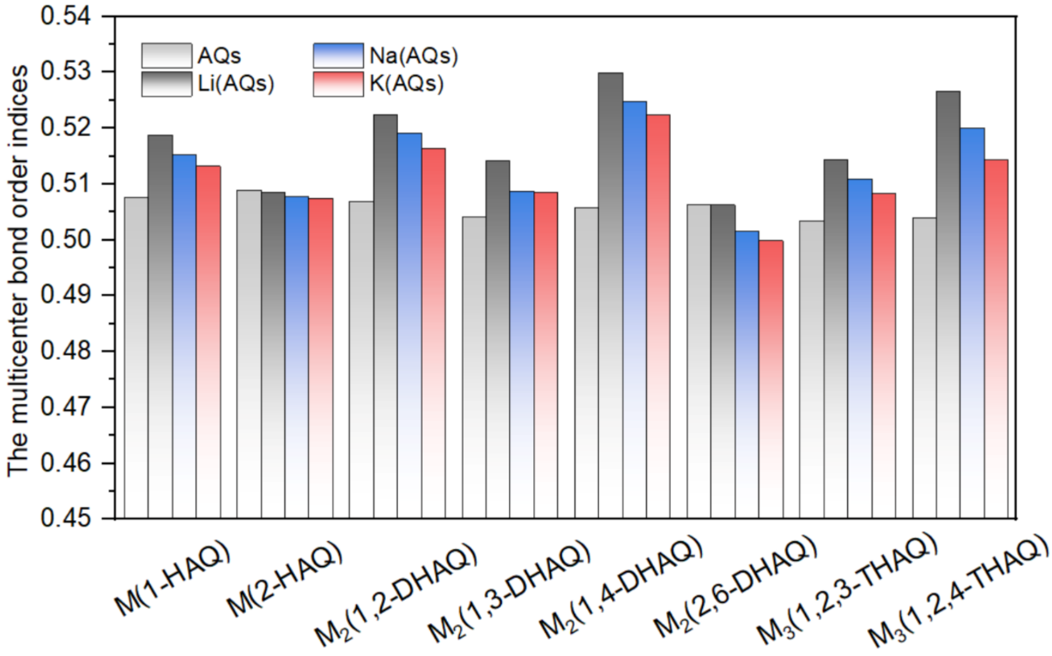

Figure S4. Multicenter bond indices of AQs and M(AQs).

The definition of multicenter bond index is based on:

$$I_{ABCDEF\ldots K} = \sum_{a\in A}\sum_{b\in B}\sum_{c\in C}\cdots\sum_{k\in K}[(PS)_{ab}(PS)_{bc}(PS)_{cd}\ldots(PS)_{ka}] \quad (8)$$

Where P is the single electron density matrix, S is the overlap matrix, a, b, c... represents the basis function number, A, B, C... represent the atomic number of the ring and these atoms are adjacent to each other according to their connection in the skeleton.[17,18]Usually a higher I value indicates a higher aromaticity. As illustrated in Table 2, the I value of AQs increases after metalation, indicating an increased aromaticity.

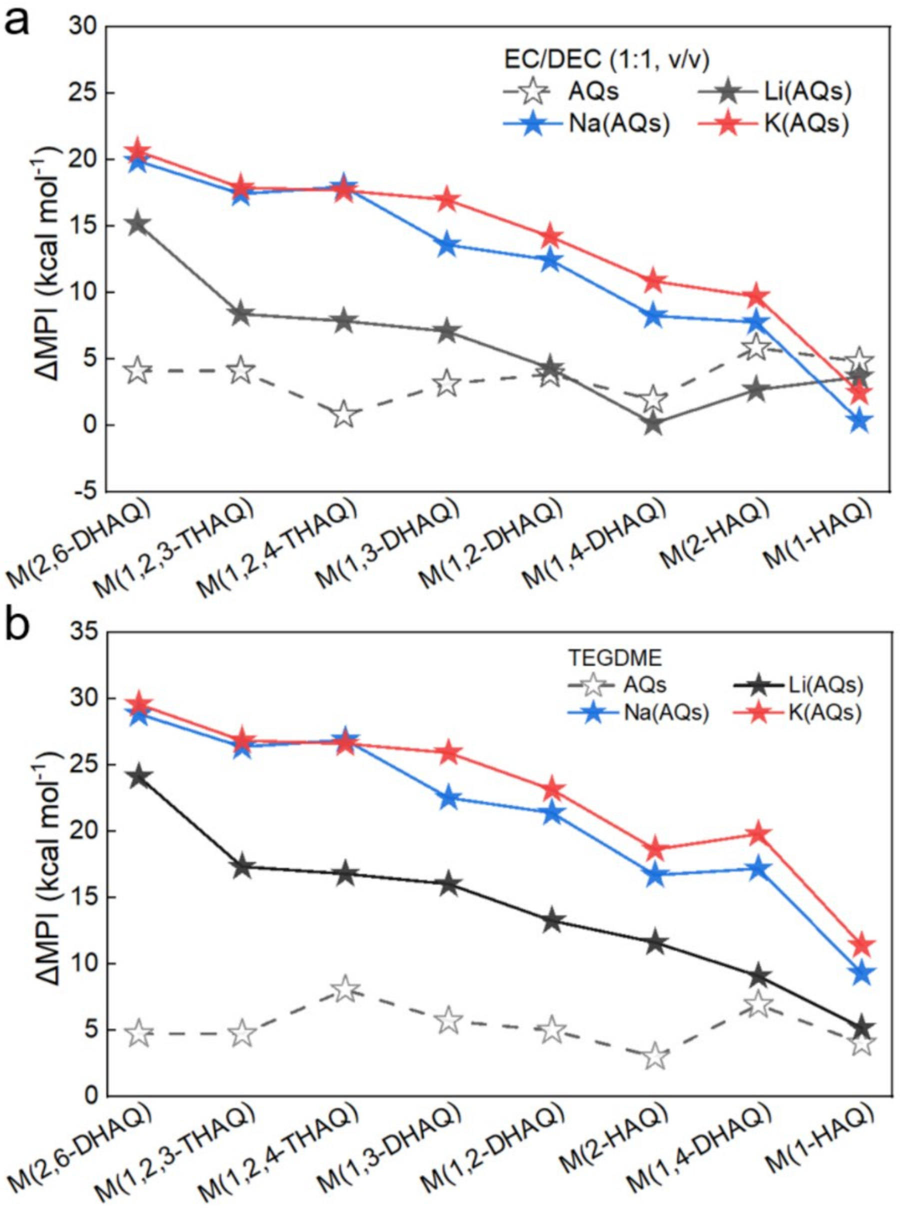

Figure S5. Difference in molecular polarity index (ΔMPI) between AQs/(M)AQs and EC/DEC (1:1, v/v) or TEGDME.

With the exception of M(1-HAQ), Li(2-HAQ), and Li_2(1,4-DHAQ), the ΔMPI values for M(AQs) in EC/DEC are higher than those of the corresponding AQs, indicating that the dissolution of AQs in EC/DEC can be generally suppressed after metalation. By contrast, the ΔMPI of all examined AQs in TEGDME increases after metalation. It can also be seen from Figure S5 that ΔMPI increases more significantly in TEGDME than in EC/DEC.

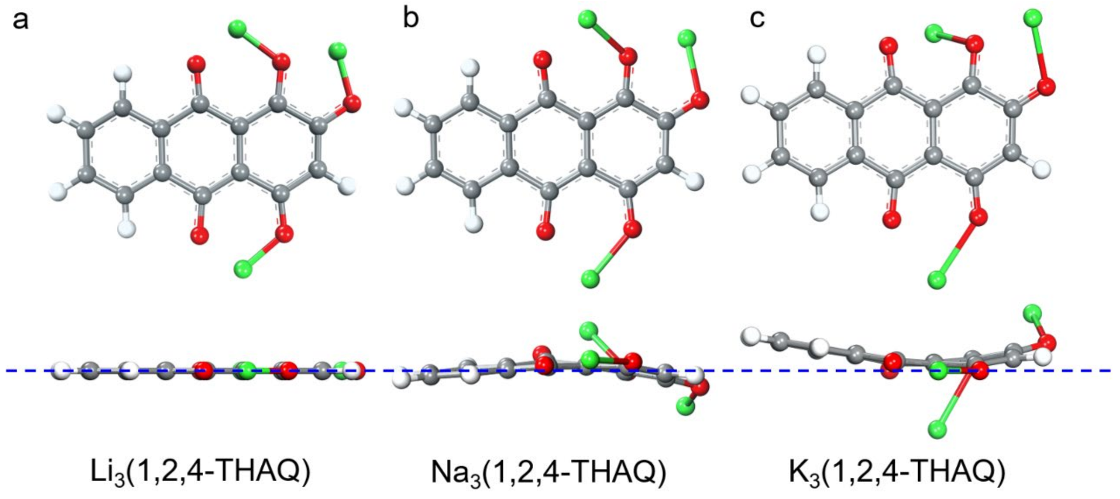

Figure S6. Structures of alkali-metalated 1,2,4-THAQs. Front and side views of the structures of (a) Li_3(1,2,4-THAQ), (b) Na_3(1,2,4-THAQ), and (c) K_3(1,2,4-THAQ), respectively.

Obvious deformation can be found for Na_3(1,2,4-THAQ) and K_3(1,2,4-THAQ), which influences their polarity at different extent.

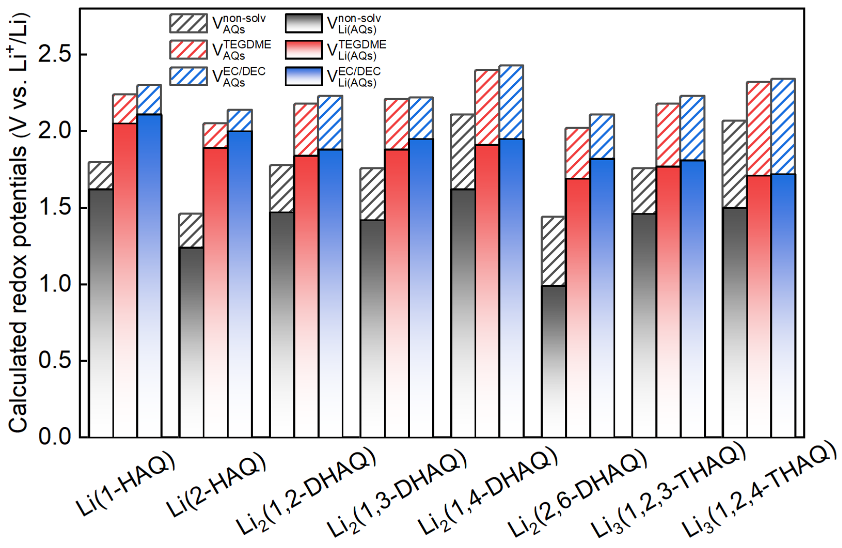

Figure S7. Calculated redox potentials of AQs and Li(AQs) in different electrolyte conditions, including solvent-free conditions, EC/DEC (1:1, v/v), and TEGDME.

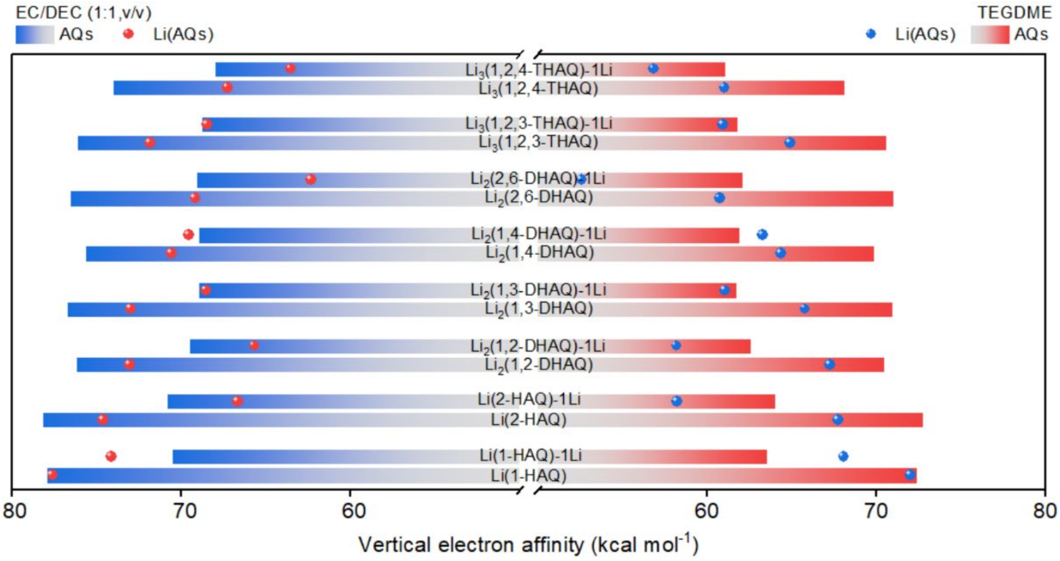

Figure S8. Vertical electron affinity of AQs, AQs-1Li, Li(AQs), and Li(AQs)-1Li in EC/DEC (1:1, v/v), and TEGDME.

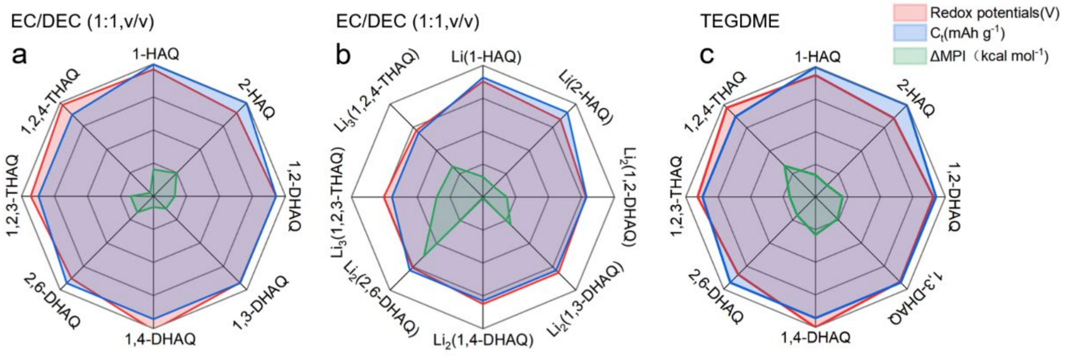

Figure S9. Radar plots of the electrochemical properties of proposed molecules.

Electrochemical properties of (a) AQs and (b) Li(AQs) in EC/DEC. (c) Electrochemical properties of AQs in TEGDME.

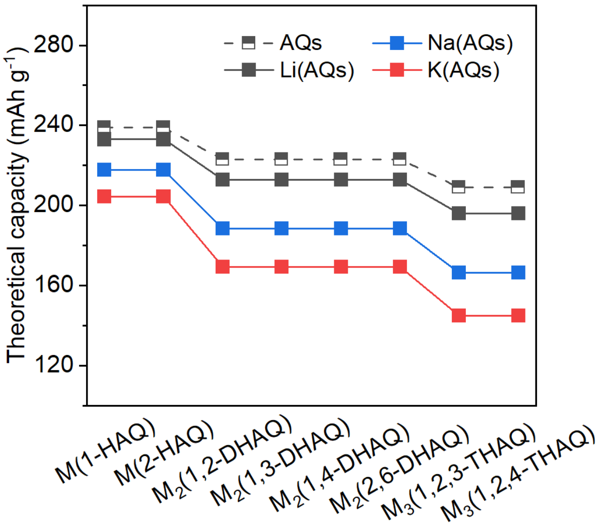

Figure S10. Theoretical capacities of AQs and their alkali-metalated salts.

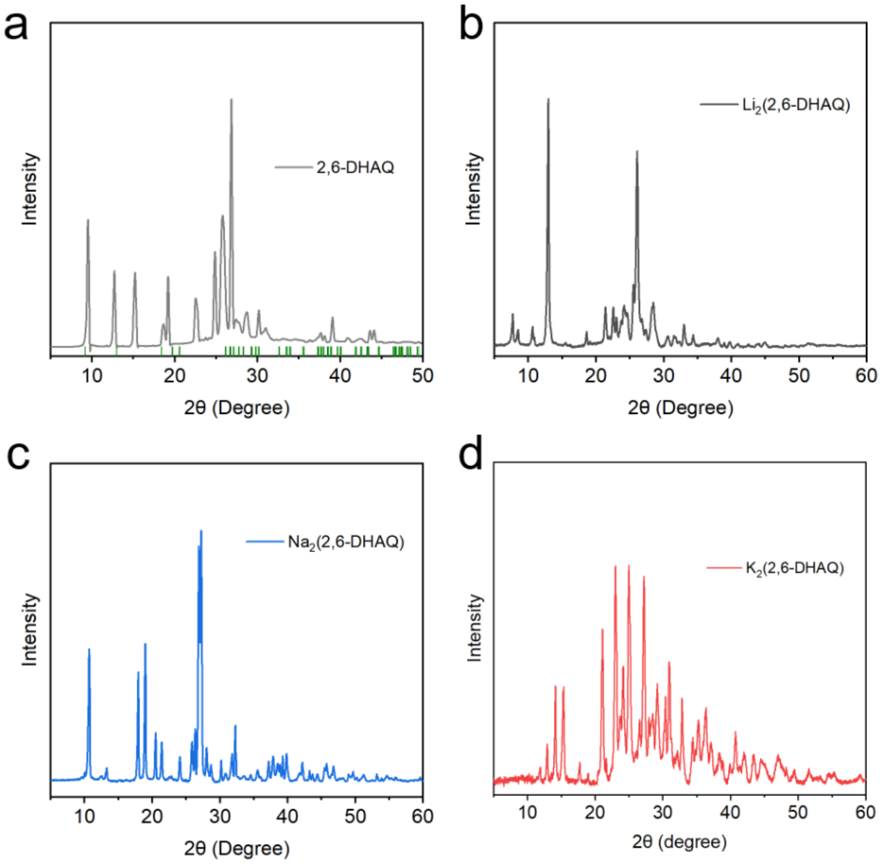

Figure S11. X-ray diffraction (XRD) pattern of 2,6-DHAQ and M_2(2,6DHAQ).

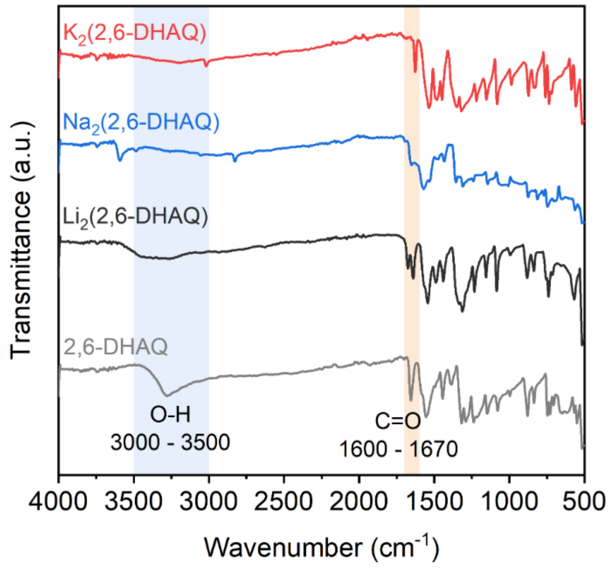

Figure S12. Fourier transform infrared spectroscopy (FT-IR) of 2,6-DHAQ and M_2(2,6DHAQ).

After metalation, the peak originated from the O-H stretching (3000 – 3500 cm^{-1}) disappeared. Meanwhile, the stretching vibration of C=O bond maintains between 1600 and 1670 cm^{-1}, demonstrating the inertness of carbonyl groups during the metalation process.

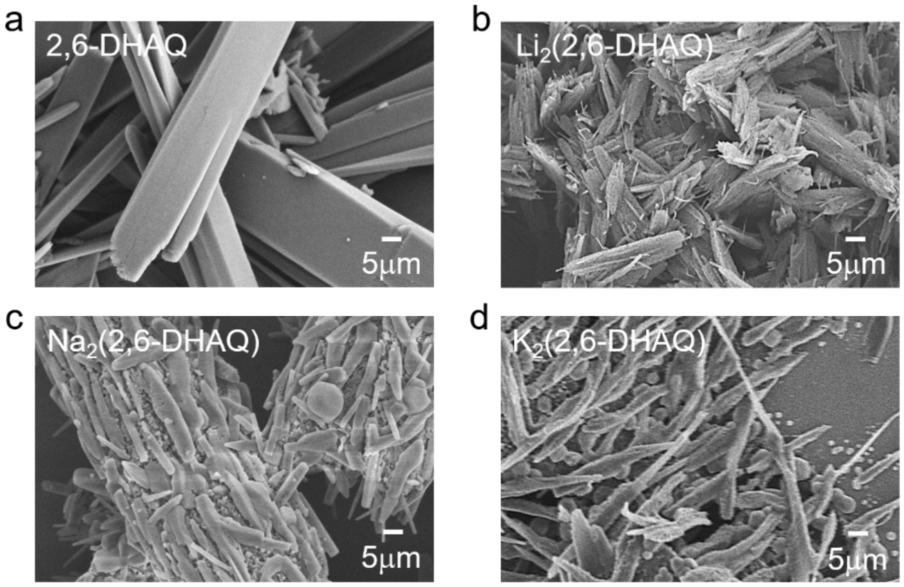

Figure S13. Scanning electron microscopy (SEM) images. SEM images of (a) 2,6-DHAQ, (b) Li_2(2,6DHAQ), (c) Na_2(2,6DHAQ), and (d) K_2(2,6DHAQ).

The crystallinity and morphology of Li_2(2,6-DHAQ), Na_2(2,6-DHAQ) and K_2(2,6-DHAQ) are given in **Figure S11** and **S13**. After metalation, the particle size of 2,6-DHAQ is reduced. It is shown in **Figure S13** that 2,6-DHAQ transforms from columnar to a needle-like morphology with reduced particle size after lithiation. After sodiation and potassiation, the particle size of 2,6-DHAQ is further reduced, resulting in the formation of K_2(2,6-DHAQ) nanoribbons. As K_2(2,6-DHAQ) exhibits a larger specific surface area than L_2(2,6-DHAQ) and Na_2(2,6-DHAQ), the exposed active materials will be more accessible to electrolyte and Li ions during charging/discharging process.

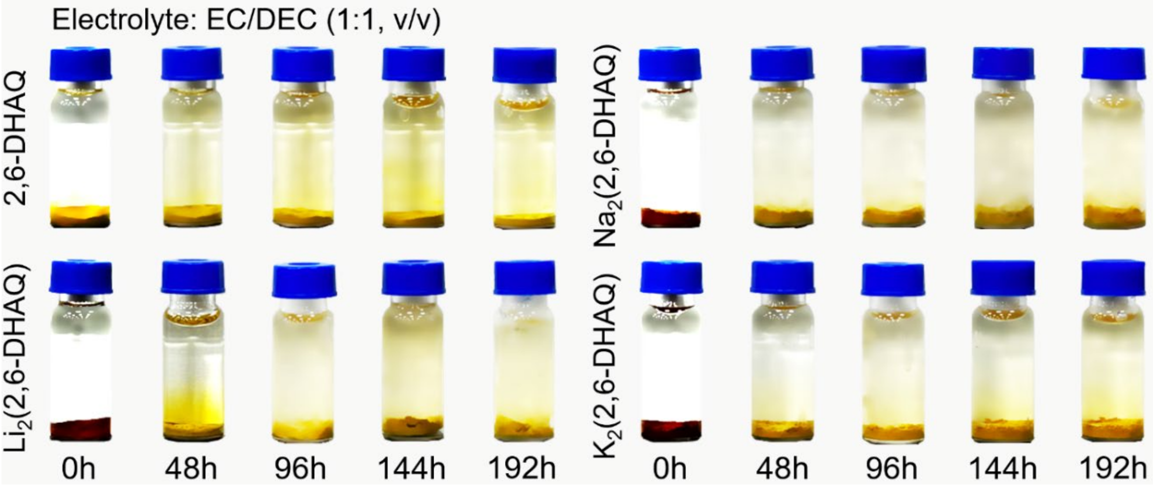

Figure S14. Photographs of solubility test.

Photographs of 2,6-DHAQ and M_2(2,6-DHAQ) dissolved in the electrolyte solution of EC/DEC (1:1) with 1M $LiPF_6$ for 48, 96, 144, and 192 hours, respectively.

The dissolution of 2,6-DHAQ in TEGDME-(1M)$LiPF_6$ is more pronounced than that in EC/DEC(1:1)-(1M)$LiPF_6$, because the polarity of 2,6-DHAQ is closer to that of TEGDME (**Figure 1b**). After metalation, Na_2(2,6-DHAQ) and K_2(2,6-DHAQ) show more reduced crystal size than that of Li_2(2,6-DHAQ). As a result, the more exposed surface area of Na_2(2,6-DHAQ) and K_2(2,6-DHAQ) than Li_2(2,6-DHAQ) leads to a more facile dissolution at the beginning in real practice.

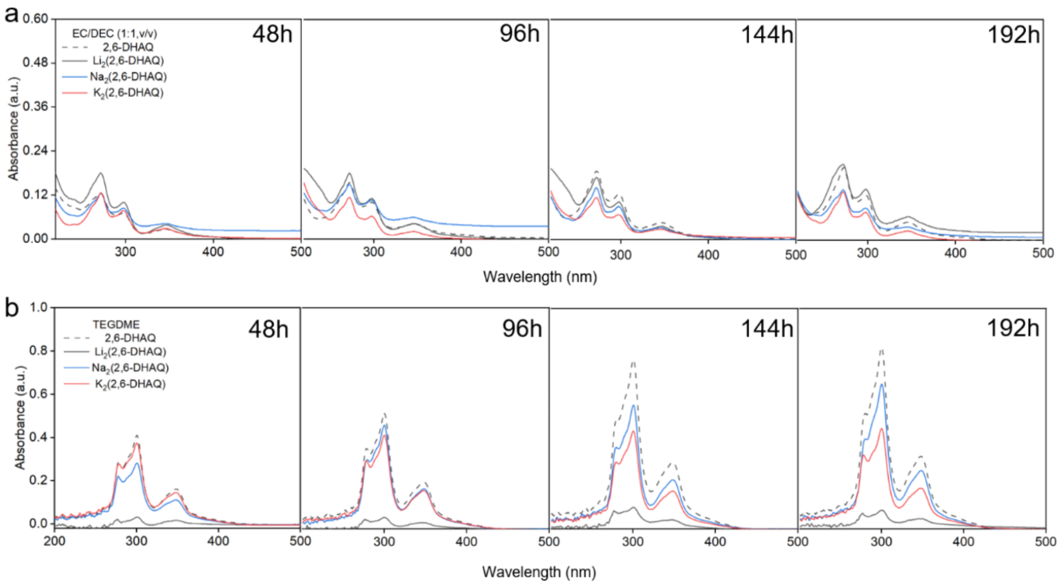

Figure S15. UV-vis spectra for solubility measurement. UV-vis spectra of (a) 2,6-DHAQ and M_2(2,6-DHAQ) in the solution of (1M)$LiPF_6$-EC/DEC (1:1, v/v) and (b) (1M)$LiPF_6$-TEGDME for 48, 96, 144, and 192 hours, respectively.

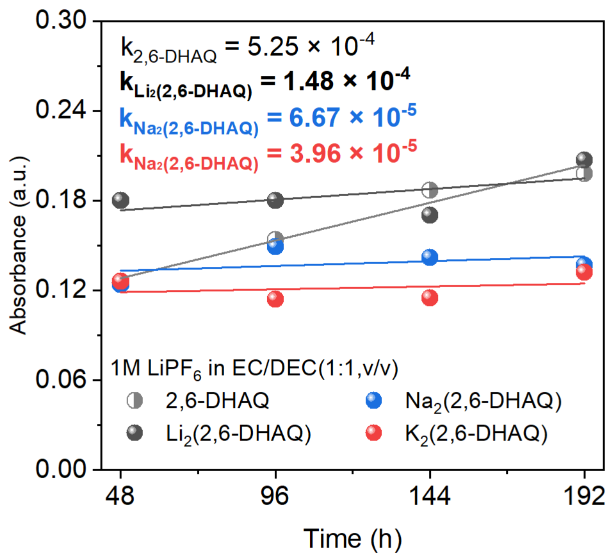

Figure S16. Highest UV-vis absorption peaks as function of exposure time and their linear fits for 2,6-DHAQ and M_2(2,6-DHAQ) after soaking in solution of EC/DEC(1:1)-(1M)$LiPF_6$ for 48, 96, 144, and 192 hours.

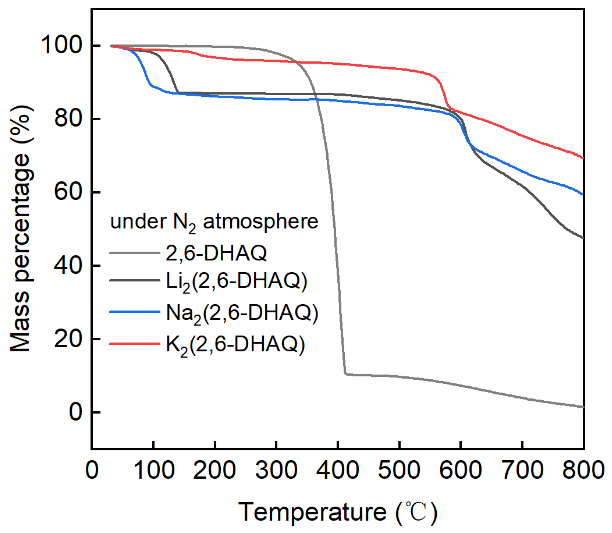

Figure S17. Test of thermal stability.

Thermogravimetric (TG) curves of 2,6-DHAQ and prepared Li_2(2,6-DHAQ), Na_2(2,6-DHAQ), and K_2(2,6-DHAQ) under a N_2 atmosphere at a heating rate of 5 ℃ min^{-1}.

It can be observed that K_2(2,6-DHAQ) exhibits the best thermal stability, thereby favoring its application in LIBs.

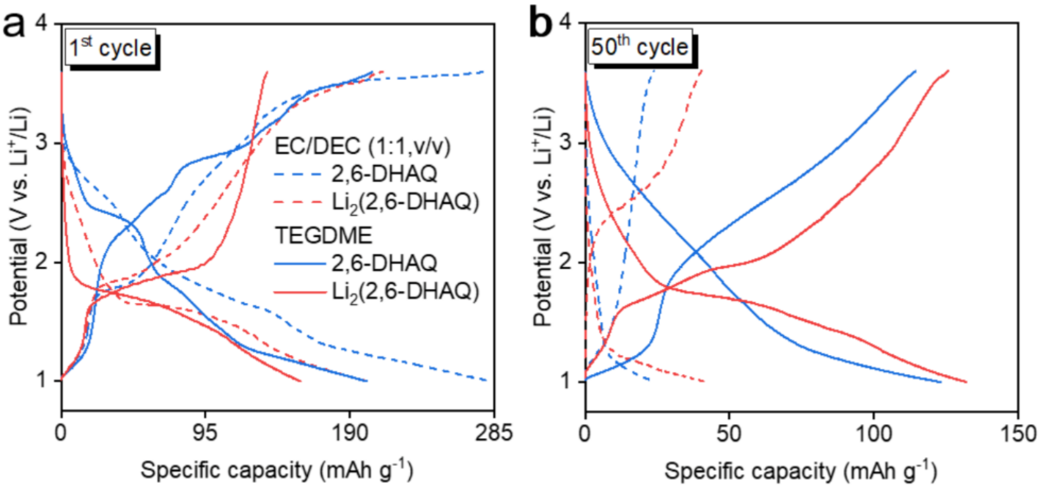

Figure S18. Charge-discharge profiles for 2,6-DHAQ and Li_2(2,6-DHAQ) in TEGDME-(1M)$LiPF_6$ and EC/DEC(1:1)-(1M)$LiPF_6$ at 0.05C for the first and 50th cycle.

As demonstrated in **Figure S18**, metalated AQs still exhibited a better electrochemical performance than the original AQs, even at a small current (0.05C).

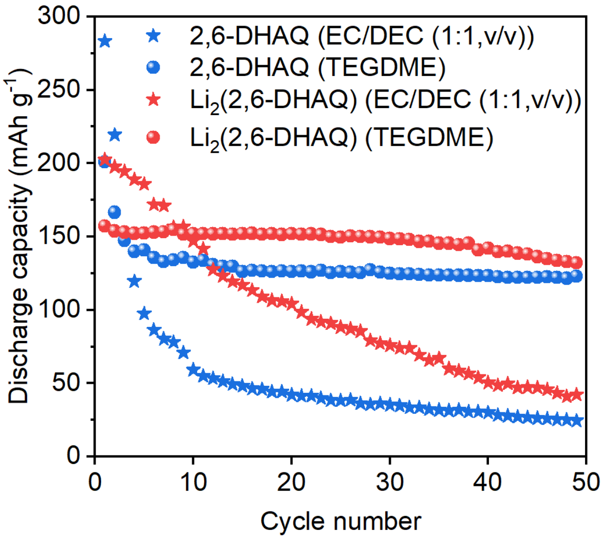

Figure S19. Cycling performance of 2,6-DHAQ and Li_2(2,6-DHAQ) in two electrolytes at 0.05 C.

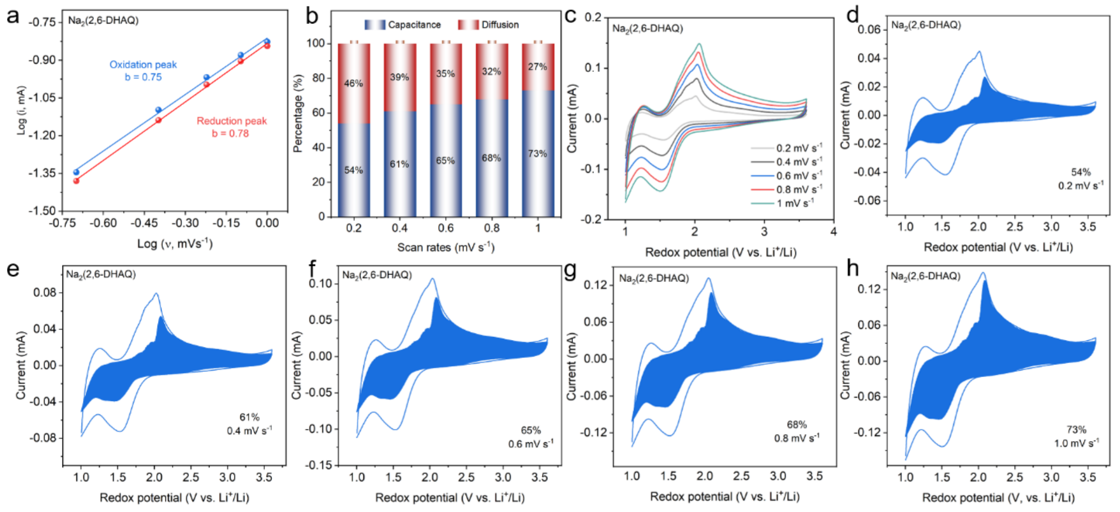

Figure S20. Quantitative analysis of Li-storage behavior using Na2(2,6-DHAQ) as redox-active material. (a) b values of cathodic/anodic peaks. (b) Capacitive and diffusion contribution of the Li^+ ion intercalation and deintercalation process of the Na2(2,6-DHAQ) electrode at multiple scan rates of 0.2–1.0 mV s^{-1}. (c) CV curves of Na2(2,6-DHAQ) at 0.2 -1 mVs^{-1}. Contribution of capacitance process at various scan rates of (d) 0.2 mV s^{-1}, (e) 0.4 mV s^{-1}, (f) 0.6 mV s^{-1}, (g) 0.8 mV s^{-1}, and (h) 1.0 mV s^{-1}, respectively.

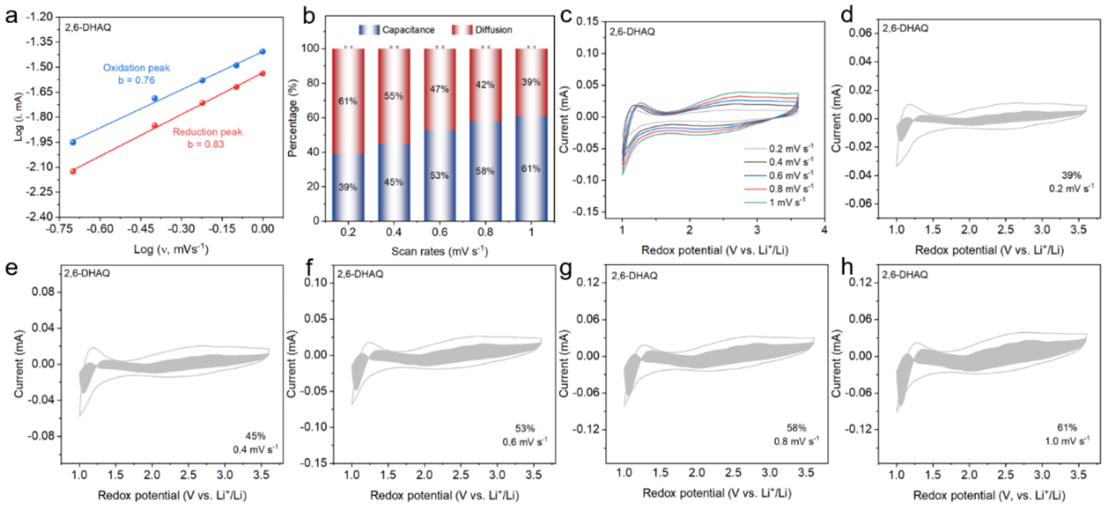

Figure S21. Quantitative analysis of Li-storage behavior using 2,6-DHAQ as redox-active material. (a) b values of cathodic/anodic peaks. (b) Capacitive and diffusion contribution of the Li^+ ion intercalation and deintercalation process of the 2,6-DHAQ electrode at multiple scan rates of 0.2 – 1.0 mV s^{-1}. (c) CV curves of 2,6-DHAQ at 0.2 -1 mVs^{-1}. Contribution of capacitance process at various scan rates of (d) 0.2 mV s^{-1}, (e) 0.4 mV s^{-1}, (f) 0.6 mV s^{-1}, (g) 0.8 mV s^{-1}, and (h) 1.0 mV s^{-1}, respectively.

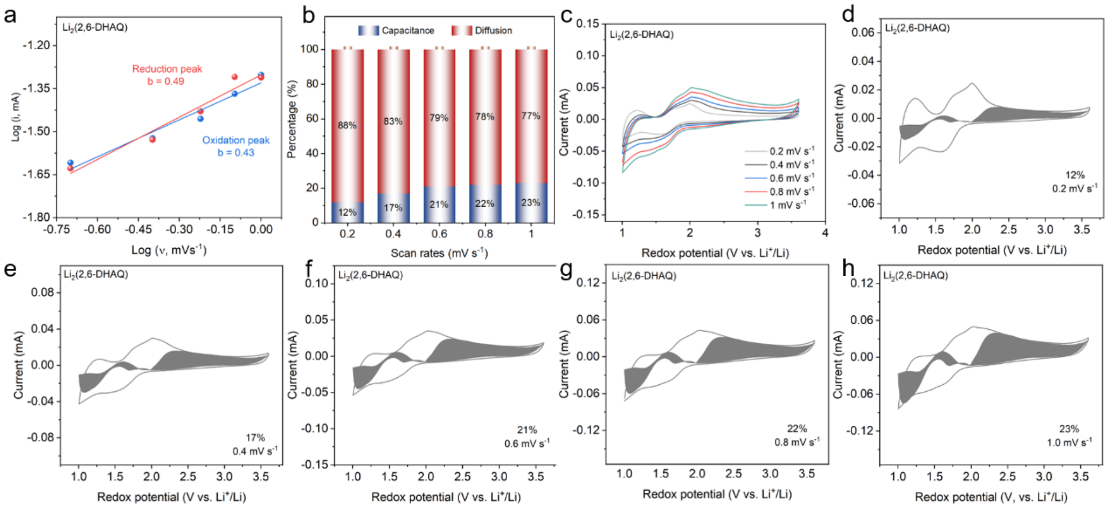

Figure S22. Quantitative analysis of Li-storage behavior using Li_2(2,6-DHAQ) as redox-active material. (a) b values of cathodic/anodic peaks. (b) Capacitive and diffusion contribution of the Li^+ ion intercalation and deintercalation process of the Li_2(2,6-DHAQ) electrode at multiple scan rates of 0.2–1.0 mV s^{-1}. (c) CV curves of Li_2(2,6-DHAQ) at 0.2 -1 mVs^{-1}. Contribution of capacitance process at various scan rates of (d) 0.2 mV s^{-1}, (e) 0.4 mV s^{-1}, (f) 0.6 mV s^{-1}, (g) 0.8 mV s^{-1}, and (h) 1.0 mV s^{-1}, respectively.

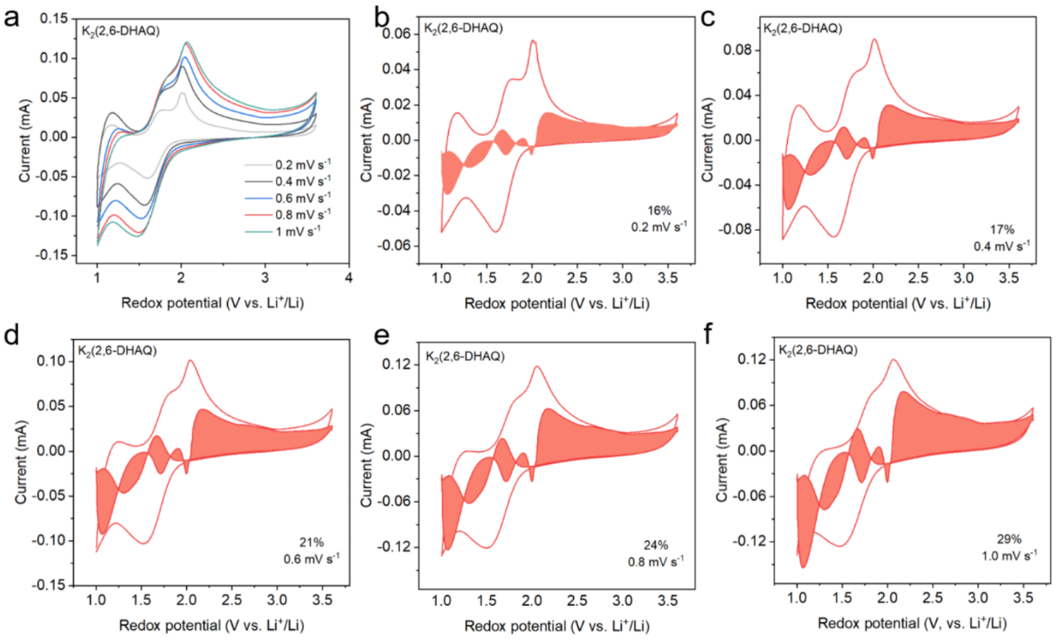

Figure S23. Quantitative analysis of Li-storage behavior using K_2(2,6-DHAQ) as redox-active material. (a) CV curves of K_2(2,6-DHAQ) at 0.2 - 1 mVs^{-1}. Contribution of capacitance process at various scan rates of (b) 0.2 mV s^{-1}, (c) 0.4 mV s^{-1}, (d) 0.6 mV s^{-1}, (e) 0.8 mV s^{-1}, and (f) 1.0 mV s^{-1}, respectively.

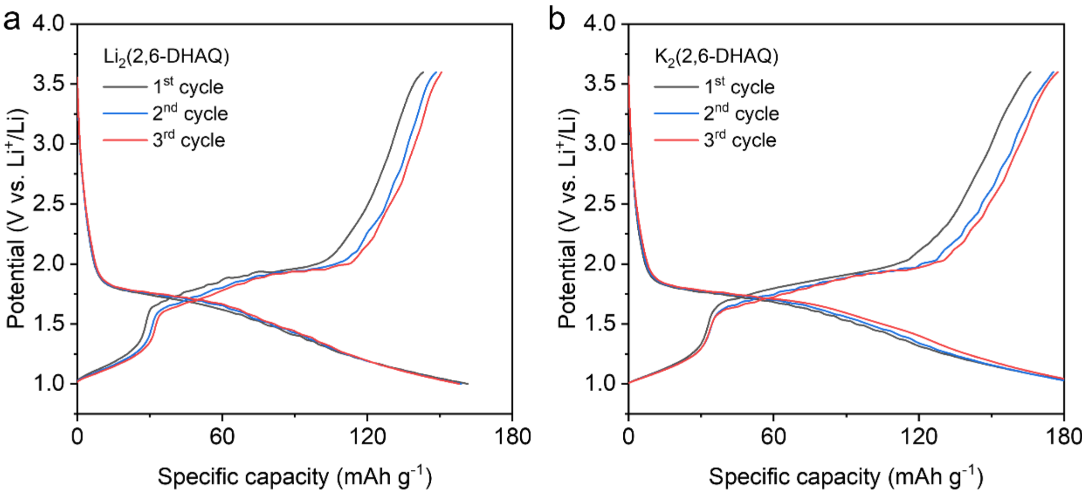

Figure S24. The charge-discharge profiles of Li_2(2,6-DHAQ) and K_2(2,6-DHAQ) for the first three cycles at 0.1 C.

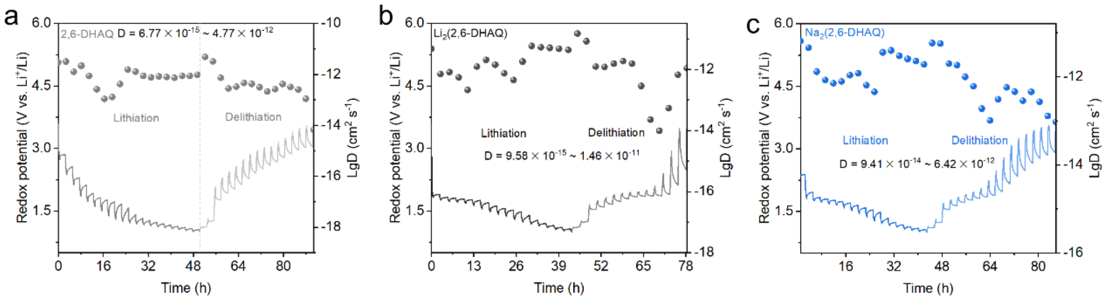

Figure S25. GITT curves and the values of D_{Li+} of 2,6-DHAQ and M_2(2,6-DHAQ) cathode in half cell. (a) 2,6-DHAQ, (b) Li_2(2,6-DHAQ), (c) Na_2(2,6-DHAQ).

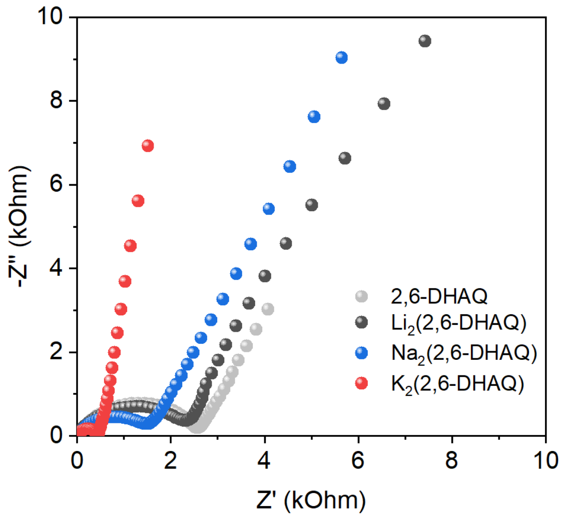

Figure S26. The Electrochemical Impedance Spectroscopy (EIS) curves of M_2(2,6-DHAQ) and 2,6-DHAQ.

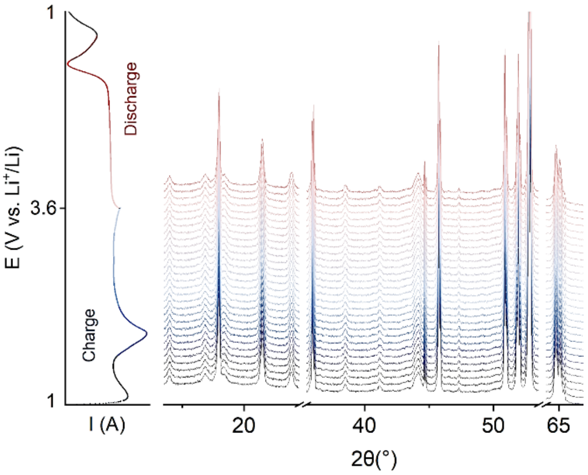

Figure S27. In-situ XRD patterns of K_2(2,6-DHAQ) cathode during the CV measurement at the scan rate of 0.5 mV s^{-1} in a half-cell.

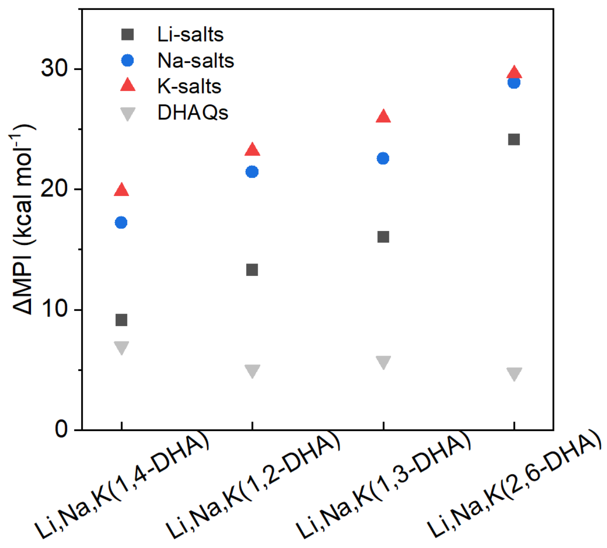

Figure S28. ΔMPI values of 1,4-DHAQ, 1,2-DHAQ, 1,3-DHAQ, 2,6-DHAQ, and corresponding alkali-metalated salts in TEGDME.

The black, blue, and red symbols represent the ΔMPI of lithiated, sodiated, and potassiated AQs salts, respectively.

The influence of alkali metalation on the polarity of AQ-salts varies with the position of hydroxyl groups. ΔMPI values of metalated two -OH substituted AQs follow the order of M_2(2,6-DHAQ) > M_2(1,3-DHAQ) > M_2(1,2-DHAQ) > M_2(1,4-DHAQ).

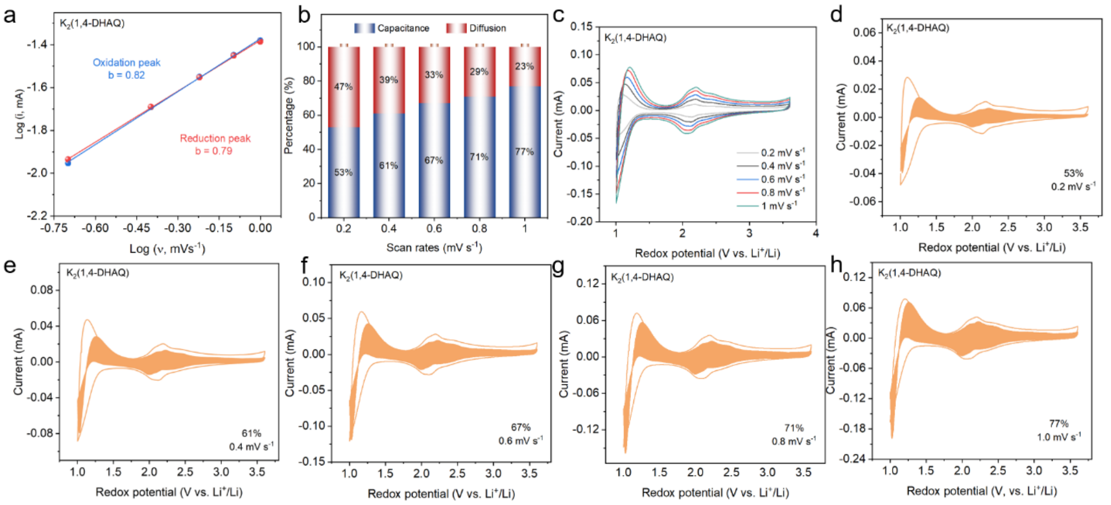

Figure S29. Quantitative analysis of Li-storage behavior using K_2(1,4-DHAQ) as redox-active material. (a) b values of cathodic/anodic peaks during CV measurement at scan rates of 0.2–1.0 mV s^{-1}. (b) Capacitive and diffusion contribution of the Li^+ ion intercalation and deintercalation process of the K_2(1,4-DHAQ) electrode at multiple scan rates. (c) CV curves of K_2(1,4-DHAQ) at 0.2 - 1 mVs^{-1}. Contribution of capacitance process at various scan rates of (d) 0.2 mV s^{-1} , (e) 0.4 mV s^{-1}, (f) 0.6 mV s^{-1}, (g) 0.8 mV s^{-1}, and (h) 1.0 mV s^{-1}, respectively.

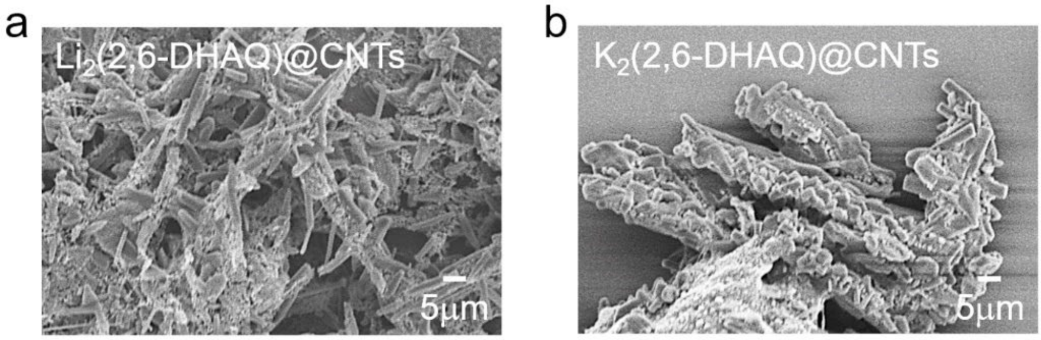

Figure S30. SEM image of K/Li_2(2,6-DHAQ)@CNTs.

As shown in Figure S29, K/Li_2(2,6-DHAQ) are coated on CNTs, and their particle size has been further reduced after the incorporation of CNTs.

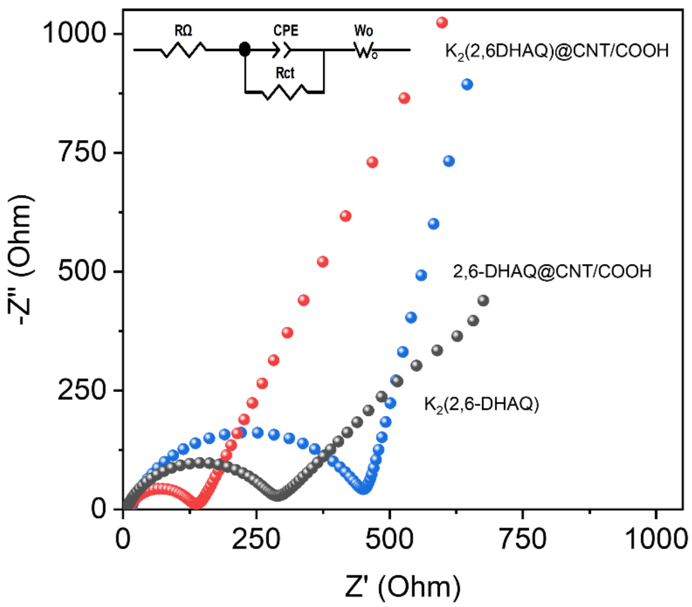

Figure S31. The Electrochemical Impedance Spectroscopy (EIS) curves of K_2(2,6-DHAQ)@CNT/COOH, 2,6-DHAQ@CNT/COOH and K_2(2,6-DHAQ). Inset: Equivalent circuit of these materials.

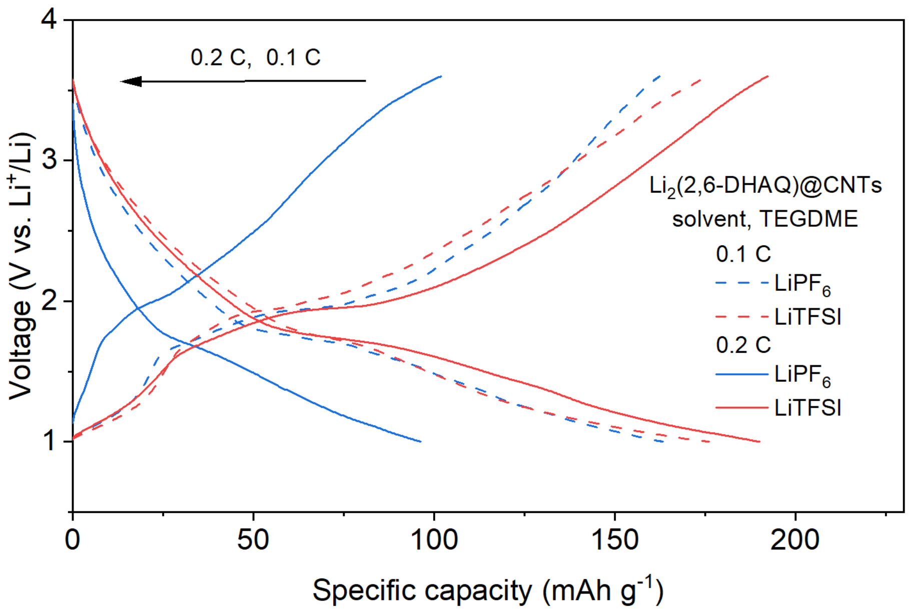

Figure S32. Charge-discharge profiles of Li_2(2,6-DHA)@CNTs at 0.1 and 0.2C in TEGDME with different solutes (1M LiTFSI and $LiPF_6$, respectively).

It can be found in Figure S31 that when using LiTFSI as the electrolyte solute, the rate performance of Li_2(2,6-DHAQ)@CNTs can be significantly improved. The capacity of Li_2(2,6-DHAQ)@CNTs in (1M) LiTFSI-TEGDME is comparable at 0.1C and 0.2C.

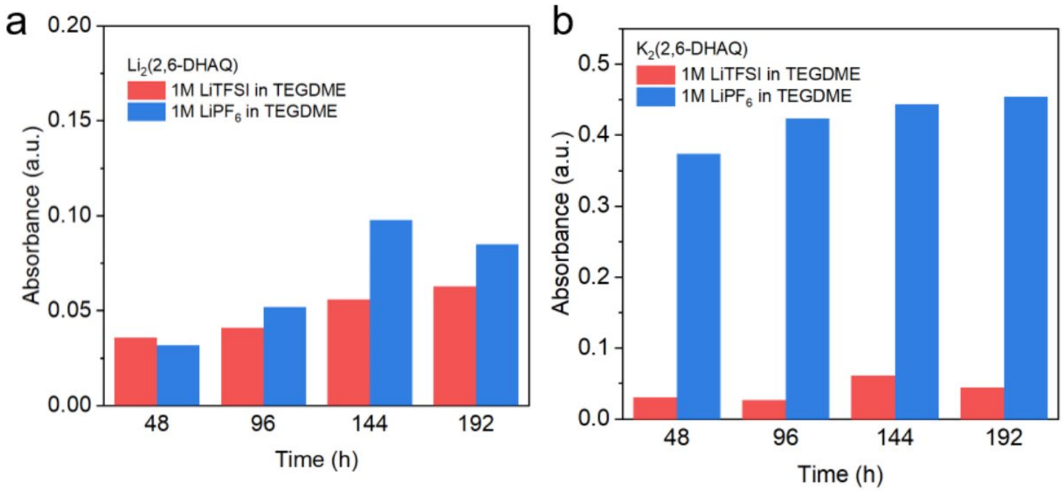

Figure S33. Highest UV-vis absorption peaks of Li/K_2(2,6-DHAQ) when soaked in different electrolytes for 48, 96, 144, 192 h.

By comparing highest UV-vis absorption peaks of Li/K_2(2,6-DHAQ) in two electrolytes consisting of different solutes (LiTFSI and $LiPF_6$), it can be found that LiTFSI is favorable for Li/K_2(2,6-DHAQ) in the half-cell system.

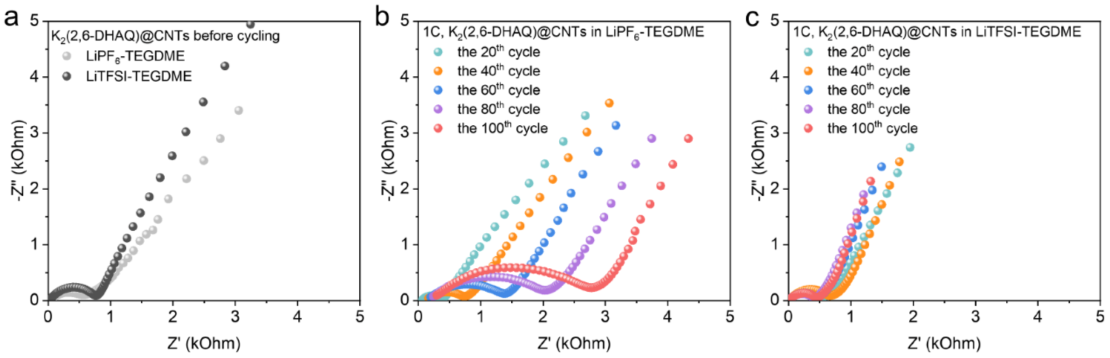

Figure S34. In situ EIS plots of K_2(2,6-DHAQ)@CNTs before and after cycling in TEGDME-$LiPF_6$/LiTFSI, respectively.

As demonstrated by Figure S33a, the initial resistance of K_2(2,6-DHAQ)@CNTs in LiPF6- and LiTFSI-TEGDME is comparable. However, the resistance of K_2(2,6-DHAQ)@CNTs in $LiPF_6$-TEGDME increases gradually with cycling, while that of K_2(2,6-DHAQ)@CNTs in LiTFSI-TEGDME hardly changes. Therefore, LiTFSI-TEGDME provides higher interfacial stability than that of T $LiPF_6$-TEGDME, rationalizing the good long-term electrochemical performance of K_2(2,6-DHAQ)@CNTs in LiTFSI-TEGDME.

Table S1. HL energy gap (eV) of AQs and M(AQs).

AQs	HL	Li(AQs)	HL	Na(AQs)	HL	K(AQs)	HL
1-HAQ	3.89	Li(1-HAQ)	2.96	Na(1-HAQ)	2.86	K(1-HAQ)	2.82
2-HAQ	3.93	Li(2-HAQ)	3.35	Na(2-HAQ)	2.67	K(2-HAQ)	2.95
1,2-DHAQ	3.72	Li_2(1,2-DHAQ)	2.39	Na_2(1,2-DHAQ)	2.13	K_2(1,2-DHAQ)	2.30
1,3-DHAQ	3.83	Li_2(1,3-DHAQ)	2.97	Na_2(1,3-DHAQ)	2.48	K_2(1,3-DHAQ)	2.44
1,4-DHAQ	3.50	Li_2(1,4-DHAQ)	2.54	Na_2(1,4-DHAQ)	2.45	K_2(1,4-DHAQ)	2.40
2,6-DHAQ	3.87	Li_2(2,6-DHAQ)	3.31	Na_2(2,6-DHAQ)	2.54	K_2(2,6-DHAQ)	2.86
1,2,3-THAQ	3.71	Li_3(1,2,3-THAQ)	2.46	Na_3(1,2,3-THAQ)	1.85	K_3(1,2,3-THAQ)	1.87
1,2,4-THAQ	3.49	Li_3(1,2,4-THAQ)	2.58	Na_3(1,2,4-THAQ)	1.94	K_3(1,2,4-THAQ)	2.24

Table S2. Multicenter bond indices (I) of AQs and M(AQs).

AQs	I_{AQs}	Li(AQs)	$I_{Li(AQs)}$	Na(AQs)	$I_{Na(AQs)}$	K(AQs)	$I_{K(AQs)}$
1-HAQ	0.508	Li(1-HAQ)	0.519	Na(1-HAQ)	0.515	K(1-HAQ)	0.513
2-HAQ	0.509	Li(2-HAQ)	0.509	Na(2-HAQ)	0.508	K(2-HAQ)	0.508
1,2-DHAQ	0.507	Li_2(1,2-DHAQ)	0.522	Na_2(1,2-DHAQ)	0.519	K_2(1,2-DHAQ)	0.516
1,3-DHAQ	0.504	Li_2(1,3-DHAQ)	0.514	Na_2(1,3-DHAQ)	0.509	K_2(1,3-DHAQ)	0.509
1,4-DHAQ	0.506	Li_2(1,4-DHAQ)	0.530	Na_2(1,4-DHAQ)	0.525	K_2(1,4-DHAQ)	0.522
2,6-DHAQ	0.506	Li_2(2,6-DHAQ)	0.506	Na_2(2,6-DHAQ)	0.502	K_2(2,6-DHAQ)	0.500
1,2,3-THAQ	0.503	Li_3(1,2,3-THAQ)	0.514	Na_3(1,2,3-THAQ)	0.511	K_3(1,2,3-THAQ)	0.508
1,2,4-THAQ	0.504	Li_3(1,2,4-THAQ)	0.527	Na_3(1,2,4-THAQ)	0.520	K_3(1,2,4-THAQ)	0.514

Table S3. Molecular polarity index (MPI, kcal mol^{-1}) of AQs and alkali-metalated AQs.

AQs	MPI	Li(AQs)	MPI	Na(AQs)	MPI	K(AQs)	MPI
1-HAQ	12.03	Li(1-HAQ)	13.19	Na(1-HAQ)	17.3	K(1-HAQ)	19.4
2-HAQ	11.00	Li(2-HAQ)	19.62	Na(2-HAQ)	24.7	K(2-HAQ)	26.63
1,2-DHAQ	12.99	Li_2(1,2-DHAQ)	21.26	Na_2(1,2-DHAQ)	29.39	K_2(1,2-DHAQ)	31.15
1,3-DHAQ	13.71	Li_2(1,3-DHAQ)	24.02	Na_2(1,3-DHAQ)	30.53	K_2(1,3-DHAQ)	33.91
1,4-DHAQ	14.93	Li_2(1,4-DHAQ)	17.1	Na_2(1,4-DHAQ)	25.19	K_2(1,4-DHAQ)	27.8
2,6-DHAQ	12.74	Li_2(2,6-DHAQ)	32.11	Na_2(2,6-DHAQ)	36.84	K_2(2,6-DHAQ)	37.57
1,2,3-THAQ	12.73	Li_3(1,2,3-THAQ)	25.33	Na_3(1,2,3-THAQ)	34.36	K_3(1,2,3-THAQ)	34.83
1,2,4-THAQ	16.06	Li_3(1,2,4-THAQ)	24.79	Na_3(1,2,4-THAQ)	34.89	K_3(1,2,4-THAQ)	34.61

Table S4-1. Dielectric constant (ε) at 298.15 K and MPI (kcal mol^{-1}) values of common electrolyte solvents.

Electrolyte	Ratio by vol.	ε	MPI (kcal mol^{-1})
ethylene carbonate (EC)[8]		95.3	21.70
dimethyl carbonate (DMC)[8]		3.08	14.77
dimethoxyethane (DME)[8]		7.2	7.92
propylene carbonate (PC)[8]		64.9	19.71
ethyl methyl carbonate (EMC)[8]		2.9	13.59
diethyl carbonate (DEC)[8]		2.82	12.08
1,3-dioxolane (DOL)[8]		7.34	11.78
tetraethylene glycol dimethyl ether (TEGDME)[7]		7.78	7.96

Table S4-2. Dielectric constant (ε) at 298.15 K and MPI (kcal mol^{-1}) values of common electrolyte solvents.

Electrolyte	Ratio by vol.	ε	MPI (kcal mol^{-1})
EC:DMC	3:7	30.75	16.85
EC:DMC	1:1	49.19	18.24
EC:DEC	1:1	49.06	16.89
EC:DME	3:7	33.63	12.05
EC:DME	1:1	51.25	14.81
EC:PC	1:1	80.10	20.71
DOL:DME	1:1	7.27	9.85

Table S5. MPI values and the ΔMPI values between the solutes and the solvents.

Compounds	MPI (kcal mol^{-1})	ΔMPI (kcal mol^{-1})
water (solvent1)	23.3	
benzene (solute1)	8.0	15.4[a]
CCl_4 (solute1)	3.7	19.6[a]
ethane (solute1)	2.4	21.0[a]
methane (solute1)	2.7	20.7[a]
toluene (solute1, solvent2)	7.3	16.1[a]
1,4-benzoquinone (solute2)	13.9	6.6[b]
1,4-naphthalene (solute2)	10.9	3.6[b]
9,10-anthraquinone (solute2)	9.3	2.1[b]
9,10-phenanthrenequinone (solute2)	12.1	4.8[b]

'a' and 'b' indicate that the solvents are water and toluene, respectively. Solutes1/solutes 2 are widely recognized as being insoluble/soluble in solvent 1 (water)/solvent 2 (toluene).

Table S6. Calculated redox potentials (V) and Theoretical capacities (mAh g^{-1}) of AQs.

AQs	C_t (mAh g^{-1})	Redox potentials (V)		
		$V_{non\text{-}solvent}$	V_{TEGDME}	$V_{EC/DEC\ (1:1,\ v/v)}$
1-HAQ	239	1.80	2.24	2.30
2-HAQ	239	1.46	2.05	2.14
1,2-DHAQ	223	1.78	2.18	2.23
1,3-DHAQ	223	1.76	2.21	2.22
1,4-DHAQ	223	2.11	2.40	2.43
2,6-DHAQ	223	1.44	2.02	2.11
1,2,3-THAQ	209	1.76	2.18	2.23
1,2,4-THAQ	209	2.07	2.32	2.34

Table S7. Calculated redox potentials (V) and Theoretical capacities (mAh g^{-1}) of Li(AQs).

Li(AQs)	C_t (mAh g^{-1})	Redox potentials (V)		
		$V_{non\text{-}solvent}$	V_{TEGDME}	$V_{EC/DEC\ (1:1,\ v/v)}$
Li(1-HAQ)	233	1.62	2.05	2.11
Li(2-HAQ)	233	1.24	1.89	2.00
Li_2(1,2-DHAQ)	213	1.47	1.84	1.88
Li_2(1,3-DHAQ)	213	1.42	1.88	1.95
Li_2(1,4-DHAQ)	213	1.62	1.91	1.95
Li_2(2,6-DHAQ)	213	0.99	1.69	1.82
Li_3(1,2,3-THAQ)	196	1.46	1.77	1.81
Li_3(1,2,4-THAQ)	196	1.50	1.71	1.72

$V_{non\text{-}solvent}$, V_{TEGDME}, and $V_{EC/DEC\ (1:1,v/v)}$ are redox potentials in solvent-free, TEGDME and EC/DEC (1:1, v/v) solvent, respectively.

Table S8. Vertical electron affinity (VEA, kcal mol^{-1}) of AQs, AQ-1Li, AQ-2Li, Li(AQs), Li(AQs)-1Li and Li(AQs)-2Li in EC/DEC (1:1, v/v).

Qs	VEA (kcal mol^{-1})			Q(Li)-salts	VEA (kcal mol^{-1})		
	0	1	2		0	1	2
1-HAQ	77.92	70.51	37.41	Li(1-HAQ)	77.61	74.14	37.42
2-HAQ	78.20	70.82	38.36	Li(2-HAQ)	74.61	66.68	34.67
1,2-DHAQ	76.20	69.47	36.87	Li_2(1,2-DHAQ)	73.06	65.68	35.59
1,3-DHAQ	76.71	68.98	36.69	Li_2(1,3-DHAQ)	72.99	68.56	33.89
1,4-DHAQ	75.65	68.97	35.95	Li_2(1,4-DHAQ)	70.57	69.57	37.37
2,6-DHAQ	76.57	69.06	37.91	Li_2(2,6-DHAQ)	69.17	62.32	30.69
1,2,3-THAQ	76.11	68.77	34.91	Li_3(1,2,3-THAQ)	71.85	68.47	32.42
1,2,4-THAQ	74.03	68.02	35.55	Li_3(1,2,4-THAQ)	67.27	63.54	35.58

Table S9. Vertical electron affinity (VEA, kcal mol^{-1}) of AQs, AQ-1Li, AQ-2Li, Li(AQs), Li(AQs)-1Li and Li(AQs)-2Li in TEGDME.

Qs	VEA (kcal mol^{-1})			Q(Li)-salts	VEA (kcal mol^{-1})		
	0	1	2		0	1	2
1-HAQ	72.35	63.53	29.73	Li(1-HAQ)	72.03	68.06	30.34
2-HAQ	72.76	64.00	30.70	Li(2-HAQ)	67.73	58.23	25.56
1,2-DHAQ	70.46	62.54	29.28	Li_2(1,2-DHAQ)	67.25	58.19	28.08
1,3-DHAQ	70.95	61.73	29.07	Li_2(1,3-DHAQ)	65.79	61.06	25.47
1,4-DHAQ	69.82	61.91	28.22	Li_2(1,4-DHAQ)	64.37	63.29	30.96
2,6-DHAQ	70.99	62.06	30.33	Li_2(2,6-DHAQ)	60.73	52.59	20.22
1,2,3-THAQ	70.54	61.75	27.35	Li_3(1,2,3-THAQ)	64.90	60.94	24.79
1,2,4-THAQ	68.13	61.07	27.97	Li_3(1,2,4-THAQ)	61.03	56.83	28.72

AQs and (Li)AQs represent eight quinone derivatives and their Li-salts respectively.

Table S10. ΔMPI (kcal mol^{-1}) between alkali-metalated AQs and TEGDME.

Li(AQs)	ΔMPI	Na(AQs)	ΔMPI	K(AQs)	ΔMPI
Li(1-HAQ)	5.23	Na(1-HAQ)	9.34	K(1-HAQ)	11.44
Li(2-HAQ)	11.66	Na(2-HAQ)	16.74	K(2-HAQ)	18.67
Li_2(1,2-DHAQ)	13.30	Na_2(1,2-DHAQ)	21.43	K_2(1,2-DHAQ)	23.19
Li_2(1,3-DHAQ)	16.06	Na_2(1,3-DHAQ)	22.57	K_2(1,3-DHAQ)	25.95
Li_2(1,4-DHAQ)	9.14	Na_2(1,4-DHAQ)	17.23	K_2(1,4-DHAQ)	19.84
Li_2(2,6-DHAQ)	24.15	Na_2(2,6-DHAQ)	28.88	K_2(2,6-DHAQ)	29.61
Li_3(1,2,3-THAQ)	17.37	Na_3(1,2,3-THAQ)	26.40	K_3(1,2,3-THAQ)	26.87
Li_3(1,2,4-THAQ)	16.83	Na_3(1,2,4-THAQ)	26.93	K_3(1,2,4-THAQ)	26.65

Table S11. Electrochemical performance of K_2(2,6-DHAQ)@CNT demonstrated in this work in comparison with that of AQs reported in previous studies.

AQs/AQ-salts	Ct (mAh g^{-1})	Specific capacity (mAh g^{-1})	Cycle number	Capacity retention	TCA	Electrolytes	Percentage of active material	Ref.
nr-LiAQC/G	208	165 (0.1C)	200	88%	79%	1M $LiPF_6$-EC/EMC/DMC (1:1:1, v/v)	40%	19
DMAQ	200	146 (0.2C)	80	73%	73%	4M LiTFSI-DOL/DME (1:1, v/v)	60%	20
(AQ)CMK-3	258	174 (0.2C)	100	85%	67%	2M-DD-1%L	80%	21
LCAQ	174	70 (0.2C)	20	82%	40%	1M $LiPF_6$-PC	23%	22
$Li_2(C_{14}H_6O_4)$	213	120 (0.1C)	50	95%	56%	1M $LiPF_6$-EC/EMC/DMC (1:1:1, v/v)	80%	23
CLP	205	90 (0.05C)	50	60%	44%	1M $LiPF_6$-EC/ DMC (1:1, v/v)	80%	24
K_2(2,6-DHAQ)@CNTs	169	164 (1C)	500	100%	97%	1M LiTFSI-TEGDME	60%	This work

Here nr-LiAQC/G represents nanorod-(anthraquinone carboxylate lithium salt)/Graphene. DMAQ is the abbreviation of 2,6-dimethoxy-9,10-anthraquinone. (AQ)/CMK-3 represents 9,10-anthraquinone (AQ)/CMK-3. LCAQ represents 2,6-bis(lithiooxycarbonyl)-9,10-anthraquinone,

and CLP represents chemically lithiated purpurin. The 2M-DD-1%L electrolyte is a mixture of 2M $LiN(CF_3SO_2)_2$, DOL/DME (1:1, v/v), and 1% $LiNO_3$.

References

[1] M. J. Frisch, G. W. Trucks, H. B. Schlegel, G. E. Scuseria, M. A. Robb, J. R. Cheeseman, G. Scalmani, V. Barone, G. A. Petersson, H. Nakatsuji, X. Li, M. Caricato, A. V. Marenich, J. Bloino, B. G. Janesko, R. Gomperts, B. Mennucci, H. P. Hratchian, J. V. Ortiz, A. F. Izmaylov, J. L. Sonnenberg, D. Williams-Young, F. Ding, F. Lipparini, F. Egidi, J. Goings, B. Peng, A. Petrone, T. Henderson, D. Ranasinghe, V. G. Zakrzewski, J. Gao, N. Rega, G. Zheng, W. Liang, M. Hada, M. Ehara, K. Toyota, R. Fukuda, J. Hasegawa, M. Ishida, T. Nakajima, Y. Honda, O. Kitao, H. Nakai, T. Vreven, K. Throssell, J. A. Montgomery, Jr., J. E. Peralta, F. Ogliaro, M. J. Bearpark, J. J. Heyd, E. N. Brothers, K. N. Kudin, V. N. Staroverov, T. A. Keith, R. Kobayashi, J. Normand, K. Raghavachari, A. P. Rendell, J. C. Burant, S. S. Iyengar, J. Tomasi, M. Cossi, J. M. Millam, M. Klene, C. Adamo, R. Cammi, J. W. Ochterski, R. L. Martin, K. Morokuma, O. Farkas, J. B. Foresman, and D. J. Fox, Gaussian, Inc., Wallingford CT, 2016.

[2] T. Lu, F. Chen, *J. Comput. Chem.* **2012**, *33*, 580-592.

[3] A. D. Becke, *J. Chem. Phys.* **1993**, *98*, 5648-52.

[4] S. Grimme, S. Ehrlich, L. Goerigk, *J Comput. Chem.* **2011**, *32*, 1456-1465.

[5] L. Goerigk, S. Grimme, *J. Chem. Theory Comput.* **2011**, *7*, 291-309.

[6] C. Reichardt, *Org. Process Res. Dev.* **2007**, *11*, 105-113.

[7] C.F. Riadigos, R. Iglesias, M. A. Rivas, T. P. Iglesias, *J. Chem. Thermodynamics* **2011**, *43*, 275–283.

[8] D. S. Hall, J. Self, J.R. Dahn, *J. Phys. Chem. C* **2015**, *119*, 22322–22330.

[9] Z. Liu, T. Lu, Q. Chen, *Carbon* **2021**, *171*, 514-523.

[10] L. Neumaier, J. Schilling, A. Bardow, J. Gross, *Fluid Phase Equilib*. **2022**, *555*, 113346.

[11] B. C. Hancock, P. York, R. C. Rowe, *Int. J. Pharm*. **1997,** *148*, 1-21.

[12] J. H. Hildebrand, *Solubility of non-electrolytes* (Reinhold Publishing Corp., Chapman & Hall, Ltd., 1936).

[13] Z. Páll, M. J. Abraham, C. Kutzner, B. Hess, E. Lindahl, Tackling exascale software challenges in molecular dynamics simulations with GROMACS. In S. Markidis & E. Laure (Eds.), Solving Software Challenges for Exascale **2015**, *8759*, 3-27.

[14] L. Martínez, R. Andrade, E. G. Birgin, J. M. Martínez, *J. Comput. Chem.* **2009**, *30*, 2157-2164.

[15] T. Lu, Sobtop, Version 1.0, http://sobereva.com/soft/Sobtop.

[16] J. Gupta, C. Nunes, S. Vyas, S. Jonnalagadda, *J. Phys. Chem. B* **2011**, *115*, 2014-2023.

[17] S. Manzetti, T. Lu, *J. Phys. Org. Chem.* **2013**, *26*, 473-483.

[18] M. Giambiagi, M. Segre de Giambiagi, C. D. Santos Silva, A. Paiva de Figueiredo, *Phys. Chem. Chem. Phys*. **2000**, *2*, 3381-3392.

[19] X. Yang, H. Deng, J. Liang, J. Liang, R. Zeng, R. Zhao, Q. Chen, M. Chen, Y. Luo, S. Chou, *ACS Appl. Mater. Interfaces* **2022**, *14*, 56808−56816.

[20] J. Yang, Z. Wang, Y. Shi, P. Sun, Y Xu, *ACS Appl. Mater. Interfaces* **2020**, *12*, 7179–7185.

[21] K. Zhang, C. Guo, Q. Zhao, Z. Niu, J. Chen, *Adv. Sci*. **2015**, *2*, 1500018.

[22] A. Shimizu, H. Kuramoto, Y. Tsujii, T. Nokami, Y. Inatomi, N. Hojo, H. Suzuki, J. Yoshida, *J. Power Sources* **2014**, *260*, 211-217.

[23] R. Zeng, X. Li, Y. Qiu, W. Li, J. Yi, D. Lu, C. Tan, M. Xu, *Electrochem. commun*. **2010**, *12*, 1253-1256.

[24] A. Reddy, S. Nagarajan, P. Chumyim, S. Gowda, P. Pradhan, S. Jadhav, M. Dubey, G. John, P. Ajayan, *Sci Rep* **2012**, *2*, 960.
